# The Dynamics of Allelochemicals and Phytotoxicity in *Eisenia fetida* during the Decomposition of *Eucalyptus grandis* Litter

**DOI:** 10.3390/plants13172415

**Published:** 2024-08-29

**Authors:** Danju Zhang, Chaoyu Lv, Shaojun Fan, Yumei Huang, Na Kang, Shun Gao, Lianghua Chen

**Affiliations:** 1 Sichuan Provincial Key Laboratory of Ecological Forestry Engineering, College of Forestry, Sichuan Agricultural University, Wenjiang, Chengdu 611130, China; lvchaoyu@stu.sicau.edu.cn (C.L.); fanshaojun@stu.sicau.edu.cn (S.F.); shungao@sicau.edu.cn (S.G.); chenlh@sicau.edu.cn (L.C.); 2 College of Landscape Architecture, Sichuan Agricultural University, Wenjiang, Chengdu 611130, China; hyumei@sicau.edu.cn; 3 Shijiazhuang Zoo, Luquan, Shijiazhuang 050200, China; kn8029@163.com

**Keywords:** ecotoxicology, litter decomposition, *Eucalyptus*, *Eisenia fetida*, DNA damage

## Abstract

Allelopathy is an underlying and controversial mechanism for detrimental environmental effects in the management of *Eucalyptus* plantations. However, little attention has been paid to the dynamics of allelochemicals and phytotoxicity in soil fauna during litter decomposition. To explore the relationship between the dynamics of phytotoxicity and allelochemicals, a decomposition experiment was conducted using 4-year-old and 8-year-old *Eucalyptus grandis* litter (0, 10, 20, 30, and 45 days). The acute toxicity of *Eisenia fetida* was assessed, and a chemical analysis of the eucalyptus leaves was performed. Biochemical markers, including total protein, acetylcholinesterase (AChE) activity, and oxidative stress levels (SOD and MDA) were measured. A comet assay was used to determine DNA damage in *E. fetida* cells. The results showed that after 20–30 days of decomposition, *E. grandis* litter exhibited stronger phytotoxic effects on *E. fetida* in terms of growth and biochemical levels. After 20 days of decomposition, the weight and total protein content of *E. fetida* first decreased and then increased over time. SOD activity increased after 20 days but decreased after 30 days of decomposition before increasing again. MDA content increased after 20 days, then decreased or was stable. AChE activity was inhibited after 30 days of decomposition and then increased or stabilized with further decomposition. Soluble allelochemicals, such as betaine, chlorogenic acid, and isoquercitrin, significantly decreased or disappeared during the initial decomposition stage, but pipecolic acid significantly increased, along with newly emerging phenolic fractions that were present. More allelochemicals were released from 8-year-old litter than from 4-year-old *E. grandis* litter, resulting in consistently more severe phytotoxic responses and DNA damage in *E. fetida*. Scientific management measures, such as the appropriate removal of leaf litter in the early stages of decomposition, might help support greater biodiversity in *E. grandis* plantations.

## 1. Introduction

*Eucalyptus* species have been introduced in many countries because of their rapid growth, broad adaptability, and high productivity for use in wood, paper, and charcoal industries [1,2]. Over 5.46 million ha of *Eucalyptus* plantations have been established in southern China [3]. However, the extensive area of *Eucalyptus* plantations and frequent harvesting during a short rotation period has led to various environmental issues, such as soil degradation, reduced biodiversity, decreased productivity, and allelopathy [1,4,5]. Allelopathy is considered to be one of the key mechanisms contributing to the decreased biodiversity associated with the development of *Eucalyptus* plantations [6,7,8].

Allelopathy is the effect of chemicals released by plants or microbes on the growth of other organisms [9]. Allelopathic compounds can be actively released by plants into the environment through volatilization, leaching, root exudation, or passive production during litter or residue decomposition [10,11,12]. Litter decomposition is one of the most important allelochemical release pathways after root exudation. Allelochemicals have undergone several abiotic and biotic processes in the soil, such as sorption and polymerization by soil organic matter and clay minerals and chemical transformation and degradation, maintaining sufficient concentrations to affect plants and other biota [13,14]. The allelopathic effects of plant litter on germination and seedling growth have been studied extensively [15,16,17]. However, limited information is available on the toxic effects of allelochemicals released by plants on the soil biota. Allelochemicals entering soils can be directly toxic to soil faunal communities or indirectly toxic by changing their food resource patterns and feeding habitats by affecting the soil microbial community [12,18,19]. Allelochemicals can also exert a positive influence on the soil biota community since they can serve as a carbon for the soil microbial community. Macrofauna, such as earthworms, occupy a large amount of soil biomass and play a vital role in the turnover of soil organic matter, nutrient cycling, and plant growth [20,21]. Earthworms can facilitate litter fragmentation and aerate soils by burrowing, increasing water infiltration, transporting soil organic matter, and reducing soil erosion by bioturbation [22]. Earthworms are ideal bioindicators of soil quality because of their sensitivity to the environment and contaminants [23,24].

Leaf litter decomposition is a major ecosystem process that determines nutrient cycling, primary productivity, and soil fertility [25]. Several studies have focused on the effects of litter quality, diversity, and environment on decomposition rate and nutrient release [26,27,28]. Different litter components have different mechanisms of litter decomposition [27,28]. Most previous studies have focused on the effects of complex phenolic compounds, such as polyphenols and tannins, on their retarding effects at the late stage of litter decomposition [29,30,31]. However, allelochemicals mainly consist of simple phenolic compounds such as phenolic acids and flavones, which can be produced through litter decomposition [32]. Although the allelopathic effects of plant litter have been extensively studied, limited attention has been paid to the dynamics of allelochemicals and their kinetic phytotoxicities during decomposition. Existing studies indicate that most of the allelochemicals are released rapidly and are degraded during the initial stage of field decomposition [33,34,35,36]. The resistant and newly emerging fractions of allelochemicals produced by litter decomposition undergo several physical and biochemical processes that can either increase or decrease phytotoxicity on other biota [34]. Previous studies have indicated that the decaying litter of *Eucalyptus* inhibits the germination and growth of target plants at the early stages of decomposition; however, phytotoxicity declines as decomposition proceeds [36]. However, little information is available on the allelochemical dynamics and phytotoxicity of soil fauna during the decomposition of *Eucalyptus* litter.

*Eucalyptus* has been widely studied for its high allelochemicals content, including water-soluble phenolics and VOCs released from decomposing litter, such as benzoic acid, hydroxybenzoic acid, vanillic acid, and terpenoids [7,37]. Many previous studies have evaluated the allelopathic effects of *Eucalyptus* litter extracts on the germination and seedling growth of target plant species [16,17]. Few studies have been conducted to evaluate the phytotoxicity of *Eucalyptus* litter to soil fauna. In addition, most previous studies have been based on laboratory, and few studies have been conducted to survey the phytotoxicity of *Eucalyptus* litter during decomposition under field conditions. *Eucalyptus grandis* is one of the main introduced, fast-growing tree species used for afforestation in Southwest China and is usually managed with a short rotation period (5–7 years) [1]. However, large areas of *E. grandis* plantations have also resulted in adverse ecological effects, such as reduced biodiversity, soil degradation, and decreased productivity [4,10,38,39]. Our previous results at the study sites showed that plant and soil biodiversity in *E. grandis* remained stable or decreased over 4–5 years, followed by a significant increase with the age of the plantation. Consistently, higher levels of VOCs and phenolic allelochemicals were detected in younger *E. grandis* litter (approximately four years old). In addition, a critical increase in allelochemicals in *E. grandis* litter was observed at the eight-year-old mark [1,7]. In this study, four- and eight-year-old *E. grandis* plantations were selected to represent the critical time point for the increase in allelochemicals. *Eisenia fetida*, a common soil fauna species used in toxicological and standard tests (OECD), was selected as the target organism, as it has been found in *E. grandis* plantations at our study sites [1,40]. The purpose of this study was to address the dynamics of phytotoxicity during the decomposition of *E. grandis* litter by measuring the growth and biochemical responses (malondialdehyde [MDA], superoxide dismutase [SOD], acetylcholinesterase [AChE], and DNA damage) of *E. fetida*. In addition, the dynamics of the identified allelochemicals were evaluated during the decomposition of the eucalyptus litter. We hypothesized that (1) allelochemicals are rapidly released and degraded in the initial stages of field decomposition, influencing the phytotoxicity dynamics of litter extracts on *E. fetida*, and (2) more allelochemicals are released from 4-year-old *E. grandis* litter than from 8-year-old litter.

## 2. Materials and Methods

### 2.1. Study Site

This study was conducted at the Experimental Station of Sichuan Agricultural University, located in the western Sichuan Province (102°57′–103°04′ E, 29°55′–29°59′ N, 570–592 m altitude). The climate is subtropical, with an average annual rainfall and temperature of 1397 mm and 17.5 °C, respectively [1]. The soil is a ferralsol with an old alluvial yellow loam and a granular structure [41]. Large *E. grandis* plantations were established at various growth stages. We selected four- and eight-year-old *E. grandis* plantations; both of the two sites were larger than 5 hectares. The study sites were planted with crops before afforestation with *E. grandis*. Cropping system, intensity, and management were typical and similar in the study region. Weed control treatments were not applied after afforestation. Three sites in each age group were selected for sampling (Table 1).

### 2.2. Biodiversity Investigation

Vegetation surveys were conducted within each plot. Three 5 × 5 m quadrats containing one 1 × 1 m sub-plot were established to quantify the woody and herbaceous plants and record the species name, number of plants, and coverage of shrubs and herbaceous plants, respectively. Three 2 × 2 m plots were randomly selected, and soil macrofauna in the litter layer and 0–5 cm and 5–10 cm soil layers were hand-sorted within a sampling area of 0.25 m^2^ (50 × 50 cm). Three sampling points within each plot were selected, litter samples (10 × 10 cm) were collected, and soil samples from 0 to 5 cm and 5 to 10 cm were collected using a cylindrical sampler (5 cm in diameter, 10 cm in height). Tullgren funnels (24 h, 38 °C) were used to extract microarthropods. All the fauna specimens extracted were preserved in 75% ethanol. Individuals were counted and identified at the family level. Rhizosphere soil samples within 2 mm of the root surface of *E. grandis* were collected and stored at −80 °C for soil microbial high-throughput sequencing [40].

### 2.3. Leaf Litter and Soil Sampling

Intact and newly fallen leaves, recognizable by their color, were collected from two mature *E. grandis* plantations using litter traps (1 m × 1 m, 1 m above the ground) in June 2021. The *E. grandis* fallen leaves were air-dried and used in laboratory and field experiments. Fifty litter bags made of plastic with a 1.5 mm mesh, allowing access to soil microbes and fauna and containing 20 g of newly fallen air-dried litter, were placed on the soil surface in the plantations. The litter bags were recovered after 0, 10, 20, 30, and 45 days of *E. grandis* litter decomposition and air-dried.

Air-dried leaf litter was cut into small pieces (<5 mm). Twenty grams of the leaf litter was shaken for 24 h in 160 mL of distilled water at a mass: volume ratio of 1:8 at room temperature. The mixture was then centrifuged (Beckman Coulter, Inc., Brea, CA, USA), filtered, and diluted to concentrations of 10 mg mL^−1^, 25 mg mL^−1^, 75 mg mL^−1^, and 125 mg mL^−1^ based on the original solution. Sterilized distilled water was used as control. Natural soil samples from surface horizons (0–15 cm) were collected from abandoned croplands near *E. grandis* plantations. The soil samples were thoroughly mixed and sieved through a 2 mm mesh to measure soil physicochemical properties.

### 2.4. Earthworms

*E. fetida* was cultivated according to OECD guidelines [42]. *E. fetida* was identified according to the pictorial keys to the soil animals of China. Earthworms were reared in a container with a substrate composed of peat moss, manure, and CaCO_3_ (the pH value was adjusted to 6–7). The earthworms were fed twice per month. Adult worms with a clitellum and similar weights (300–400 mg) were selected. The earthworms were removed from the culture, rinsed with water, placed on dampened filter paper, kept in the dark at 18 °C for depuration, and acclimated to the test soil before treatment.

### 2.5. Identification of Secondary Metabolites

Ten grams of air-dried pure *E. grandis* litter samples at the initial stage and two months after decomposition from each plantation were subjected to extraction with 95 mL of 80% methanol (Thermo Fisher Scientific, Waltham, MA, USA). Before ultrasonic extraction (Zhengzhou Shengyuan Instrument Co., Ltd., Zhengzhou, China) (30 kHz for 40 min), the solutions stored in 250 mL brown flasks were shaken (Changzhou Tianrui Instrument Co., Ltd., Suzhou, China) for 24 h for a more complete extraction. The suspension was then filtered through a 0.45 μm micro-porous membrane and stored at −70 °C until subsequent ultra-performance liquid chromatography–tandem mass spectrometry (UPLC-MS/MS) analysis [32].

Metabolites of all thawed litter samples were extracted using methanol at 4 °C. Extraction solution (180 μL) from each sample and 20 μL of 100 ppm internal standards (naringenin and baicalein) were added to a 2 mL centrifuge tube. Subsequently, 20 μL of each sample was mixed with a quality control (QC) sample, and the remaining samples were subjected to UPLC-MS detection. Liquid chromatography was performed using a Thermo Ultimate 3000 system (Thermo Fisher Scientific) equipped with an AXQUTTY UPLC^®^ HSS T3 column (150 × 2.1 mm, 1.8 μm; Waters, Milford, MA, USA). Mass spectrometry was performed using a Thermo Q Exactive Focus mass spectrometer (Thermo Fisher Scientific). High-energy collision-dissociation cells were used for secondary dissociation to acquire MS/MS information, and unnecessary information was removed by dynamic exclusion.

After converting the raw data into XCMS format with Proteowizard software (V3.0.8789), the identification, filtration, and alignment of peaks were processed using the XCMS package in R (V3.3.2). To compare different magnitudes within the data, we obtained a dimensionless unit, that is, the normalized intensity, by normalizing the peak areas. All data were processed for QC to obtain reliable and high-quality metabolomic data. The peaks were matched using Metlin (http://metlin.scripps.edu, accessed on 20 August 2021), MoNA (http://mona.fiehnlab.ucdavis.edu, accessed on 20 August 2021), and a proprietary database (BioNovoGene; Suzhou, China). The OPLS-DA model was used to determine the differential metabolites (DMs) between groups based on thresholds of variable importance in projection (VIP) ≥ 1 and *p* < 0.05 [1,32].

### 2.6. Acute Toxicity

Brown glass bottles (200 mL) filled with 200 g of air-dried and sieved abandoned cropland soils were prepared. The litter extracts (20 mL) were mixed into the soil samples and stirred using glass sticks, with a range of concentrations (CK and C1-C4) as follows: 0.00, 1.00, 2.50, 7.50, and 12.50 g kg^−1^ sets for the acute toxicity tests. In total, the toxicity test involved five litter decomposition times × two forest ages × five test concentrations × three replicates = 150 bottles. Ten healthy *E. fetida* individuals were randomly placed into each bottle. These bottles were sealed with sterile, breathable sealing films. The bottles were then placed in a cabinet (Zhengzhou Shengyuan Instrument Co., Ltd.) maintained at 20 °C, with 1000 illumination and a 12/12 h photoperiod. Water was added to the samples, and the soil water content was adjusted to 30% of the water-holding capacity. Soil samples dried at 105 °C for 24 h were used to determine the soil water content. The water-holding capacity was determined as described in the ISO guidelines [43]. Water was added to ensure constant substrate water content by weighing the containers.

The mortality of the earthworms was determined after 7 and 14 days of exposure. Earthworms were sorted by hand and considered dead if they did not respond to a gentle mechanical stimulation. Living earthworms were rinsed with distilled water, dried on a filter paper, and weighed.

### 2.7. Physiological Properties

Two live *E. fetida* from each replicate of each treatment were randomly selected for the measurement of physiological properties. Normal saline (0.9%) (determination of total protein, MDA content, and SOD activity) or extracting solution (determination of AChE activity) was added, as well as the remains of the earthworms (to 1:9 weight-to-volume ratio). The mixture was then homogenized under ice-cold conditions and centrifuged at 8000 rpm for 15 min at 4 °C [44]. The supernatant was collected and stored to evaluate enzymatic activity and protein content. Physiological properties were determined using test kits following the manufacturer’s instructions. Test kits for total protein content, superoxide dismutase (T-SOD) activity, and MDA content were purchased from Nanjing Jiancheng Bioengineering Institute (Nanjing, China). Test kits for AChE) activity were purchased from Beijing Solarbio Science & Technology Co., Ltd. (Beijing, China).

Total protein content was determined using the bicinchoninic acid (BCA) method [45]. Under alkaline conditions, the protein reduces Cu^2+^ to Cu^+^, forming purple complexes with BCA. Supernatants and various reagents were pipetted into 2 mL centrifuge tubes in an orderly fashion, swirled well, and allowed to stand for 5 min. Subsequently, 200 μL was pipetted from the tubes into wells of a 96-well transparent plate. The absorbance of the microplate was measured at 562 nm (AU_562nm_) using a microplate reader [46].

SOD activity was determined using the xanthine oxidase–hydroxylamine method; O_2_^−^ was produced by the xanthine and xanthine oxidase reaction, which oxidized hydroxylamine to form nitrite and presented purple red under the action of a chromogenic agent. Color was read at 550 nm using a microplate reader [47]. MDA reacts with thiobarbituric acid at 95 °C for 80 min to form a red product, and the color was read using a spectrophotometer at 532 nm [48]. AChE catalyzes the hydrolysis of Ach to produce choline, which reacts with 5,5’-dithiobis-2-nitrobenzoic acid (DTNB) to produce TNB, and the absorbance was then measured at 412 nm using a microplate reader [49].

### 2.8. Comet Assay

Based on the following results, the effects of litter extracts on the growth and physiological properties of earthworms were stronger after 30 days of litter decomposition. Earthworms were cultivated in brown bottles treated with *E. grandis* litter extracts for 30 days of decomposition. After 14 days, earthworm coelomocytes were extracted. Three *E. fetida* individuals were selected from each bottle, rinsed with distilled water, and soaked in chilled extrusion medium (5% ethanol, 95% saline, 2.5 mg mL^−1^ Na_2_ EDTA, and 10 mg mL^−1^ guaiacol glyceryl ether, pH 7.3) for 3 min [50]. The obtained coelomocyte cells were released, washed three times in phosphate-buffered saline, and centrifuged at 4 °C for 5 min. The final cell concentration was adjusted to approximately 10^6^ cells mL^−1^ with phosphate-buffered saline.

The cell suspension was mixed with melted comet agarose (37 °C) at 1:10 (volume ratio) in an EP tube. The mixture (20 μL) was added to the wells of a 6-Well Comet Slide, covered with a coverslip, and incubated at 4 °C for 15 min under yellow light to mitigate UV-induced DNA damage. Alkaline electrophoresis was conducted in a buffer of 300 mA NaOH and 1mM Na_2_EDTA (pH > 13) for 30 min at 25 V and 300 mA, using a horizontal electrophoresis apparatus. After washing twice with distilled water for 5 min each, the slides were dehydrated in ethanol (10 min) and dried. Then, 40 μL of dyeing solution was added to each agarose spot and stained in a humidified dark box for 10 min at room temperature. A fluorescence microscope (BX41 Olympus, Tokyo, Japan) was used for observation and capture of comet images. The captured images were analyzed using Comet A 1.0 software (Cell Biolabs, San Diego, CA, USA). The obtained data of tail DNA (%), tail length, tail moment, and OTM data were utilized for the assessment of DNA damage in earthworms. OTM refers to the tail moment (distance between the center of mass of the tail and that of the head) multiplied by the percentage of DNA within the tail. It is also the most sensitive parameter reflecting the quality and quantity of DNA damage [14].

### 2.9. Statistical Analysis

The survival rate of *E. fetida* was calculated as follows:

SR = (S_0_ − D_t_)/S_0_ × 100%

where S_0_ is the initial number of earthworms, and D_t_ is the number of dead earthworms after t days.

The rate of weight inhibition of *E. fetida* was calculated as follows:

IR = (W_0_ − W_t_)/W_0_ × 100%

where W_0_ is the initial weight of earthworms and W_t_ is the weight of earthworms after t days [44].

The diversity indices were calculated as follows:

$$\mathrm{Shannon}–\mathrm{Wiener}\;\mathrm{diversity}\;\mathrm{index}\;(H)=-\sum_{i=1}^{n}P_{i}\ln P_{i}$$

Pielou evenness index (E)=H/ln⁡S

Berger–Parker index (*D_B-P_*) = *N*_max_ − *N*

$$\mathrm{Simpson}\;\mathrm{index}\;(D)=1-\sum P_{i}^{2}$$

where *N_i_* is the number of individuals in the *i*-th group, *N* is the number of individuals in all groups in the community, *N_max_* is the number of individuals of the species with the highest abundance in the sample unit, *P_i_ = N_i_/N,* and *S* is the number of groups.

All statistical analyses were performed using the SPSS software (version 25.0, SPSS Inc., Chicago, IL, USA), PAST 4.02, and R 4.3.1. Graphics were plotted using Origin 2021 and R version 4.3.1. The exposure time was taken as a within-subjects variable, and the decomposition time, age, and dose were taken as between-subjects factors to conduct repeated measurement analysis of variance and interaction analysis for all indicators. One-way analysis of variance (ANOVA) and Tukey’s test were used to analyze the significance of the differences in growth, physiological properties, and DNA damage depending on litter decomposition time and concentration. The data normality and homogeneity of the variations were tested using Shapiro–Wilk and Levene’s test, respectively. Data sets failing the tests were Ln transformed or analyzed using non-parametric tests before further analysis to help satisfy the requirement of normality and variance homogeneity. An independent sample *t*-test was used to analyze the significance of the differences in the above-mentioned properties of earthworms depending on forest age and exposure time. Analysis of similarity based on Bray–Curtis distances was used to determine differences in the composition of metabolic compounds in litter in the two aged *E. grandis* plantation stands during litter decomposition. Non-metric multidimensional scaling (NMDS) ordination was used to plot differences. RDA analysis was used to determine the correlations between the main differential potential allelochemicals in litter and understory environmental factors in the two aged plantations, such as pH, water content, organic matter, and N and P contents, and biodiversity was determined before and after two months of litter decomposition. RDA analysis was also used to determine the correlations between the growth and physiological properties of *E. fetida* and the main differential potential allelochemicals in litter.

## 3. Results

### 3.1. Soil Properties and Plant and Soil Biodiversity

The soil bulk density did not differ significantly between the two mature plantations. Soil water content and soil pH were higher in the 4-year-old stands than in the 8-year-old stands. The soil organic matter, total N, and available P contents were higher in the 8-year-old stands than in the 4-year-old stands (Table 1). Regarding woody species, the species number, density, and Shannon–Wiener index were higher in 8-year-old stands than in 4-year-old stands, whereas the Berger–Parker indices showed the opposite trend. For macrofauna and microfauna, the Shannon–Wiener index was higher in 8-year-old stands than in 4-year-old stands; however, the Berger–Parker indices of macrofauna exhibited the opposite trend (Table 2).

### 3.2. Secondary Metabolites during E. grandis Litter Decomposition

**Figure 1:**
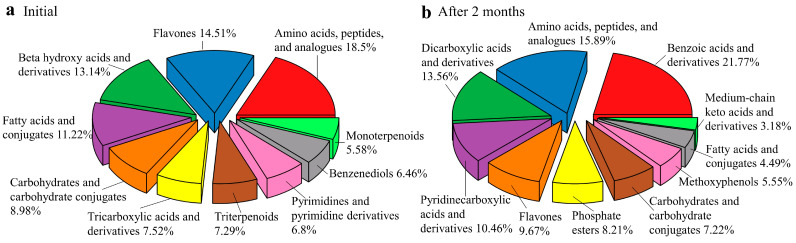
Top 10 categories of metabolites identified in litters of 4- and 8-year-old *E. grandis* at the initial decomposition stage (**a**) and two months after decomposition (**b**).

**Figure 2:**
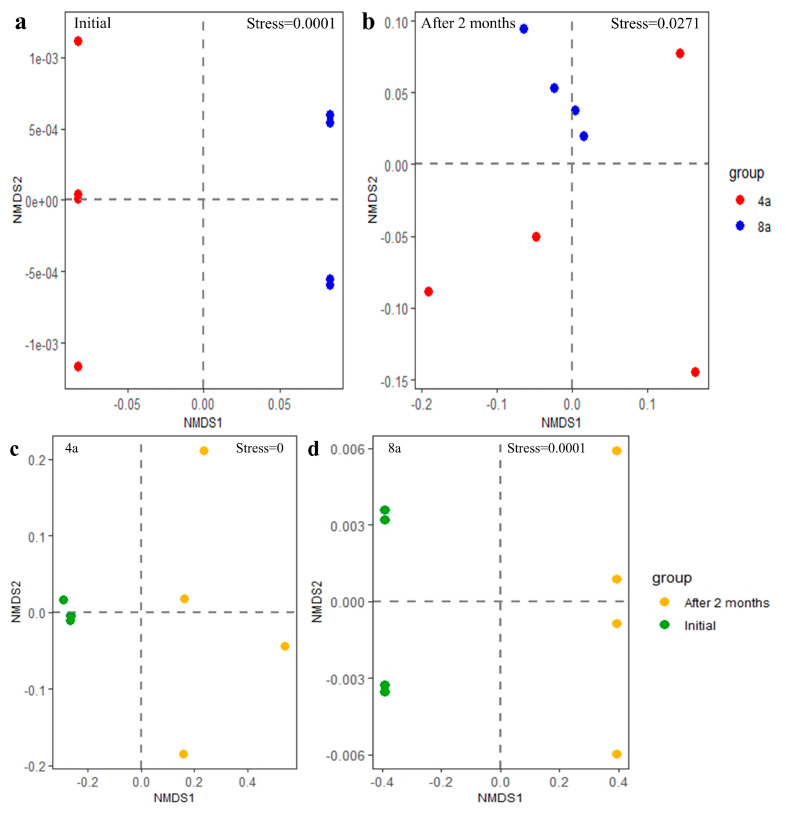
Non-metric multidimensional scale ordination for metabolites of 4- and 8-year-old *E. grandis* at the initial stage of leaf litter decomposition (**a**) and two months after decomposition (**b**); non-metric multidimensional scale ordination for metabolites at the initial stage of leaf litter decomposition and two months after decomposition at 4-year-old (**c**) and 8-year-old *E. grandis* (**d**).

A total of 328 and 381 primary and secondary metabolites, respectively, were identified in the litter of the two mature *E. grandis* plantations before and after two months of litter decomposition. The identified metabolites mainly included low-molecular-weight primary compounds, such as carbohydrates, amino acids, peptides, nucleotides and purines, cofactors, and vitamins. In addition, a wide variety of secondary metabolites, such as phenolic acids, flavones, terpenoids, alkaloids, and non-protein amino acids, were observed in *E. grandis* litter (Figure 1). The composition of metabolites in the litter varied between the two *E. grandis* plantations during litter decomposition (Figure 2).

**Figure 3:**
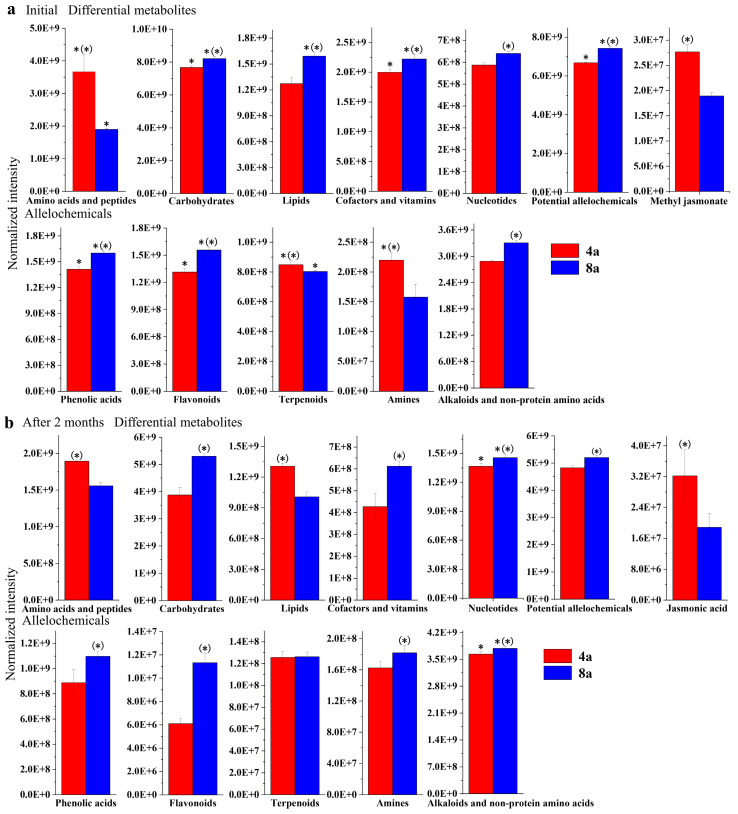
Normalization intensity of differential metabolites and potential allelochemicals in 4- and 8-year-old *E. grandis* at the initial stage of leaf litter decomposition (**a**) and two months after decomposition (**b**). * indicates significant differences between the two decomposition times; (*) indicates significant differences between the two aged *E. grandis* stands; *p* < 0.05. Error bars indicate the standard errors.

A total of 154 and 120 DMs were identified in the litter of the two mature *E. grandis* plantations during the litter decomposition, respectively. A total of 301 and 310 DMs were detected before and after two months of decomposition in the 4- and 8-year-old stands, respectively. Notably, the levels of amino acids and peptides were higher in the litter of 4-year-old *E. grandis* than in 8-year-old *E. grandis*, a pattern also observed for lipids after two months of decomposition. Conversely, other primary metabolites were more abundant in the litter of 8-year-old *E. grandis* than in the 4-year-old *E. grandis*. Among the secondary metabolites, higher levels of terpenoids and amines were observed in the initial litter of the 4-year-old than in the 8-year-old *E. grandis*. Other potential allelochemicals, such as phenolic acids, flavonoids, alkaloids, and non-protein amino acids, were more abundant in 8-year-old stands than in 4-year-old stands. A similar trend was observed for amines in the litter after two months of decomposition. Notably, no significant variation in terpenoid levels was observed between the two mature *E. grandis* after two months of decomposition. Except for lipids in the 4-year-old *E. grandis* and amines in the 8-year-old *E. grandis*, there were no significant differences during decomposition; nucleotides, alkaloids, and non-protein amino acids exhibited a significant increase after two months of litter decomposition, whereas the other primary and secondary metabolites decreased significantly after litter decomposition for the same months (Figure 3). SIMPER analysis revealed that betaine, pipecolic acid, phenol, gamma-aminobutyric acid, and the other 13 potential compounds contributed to the variation in allelochemical composition during *E. grandis* litter decomposition and between the two mature plantations (Table 3).

**Figure 4:**
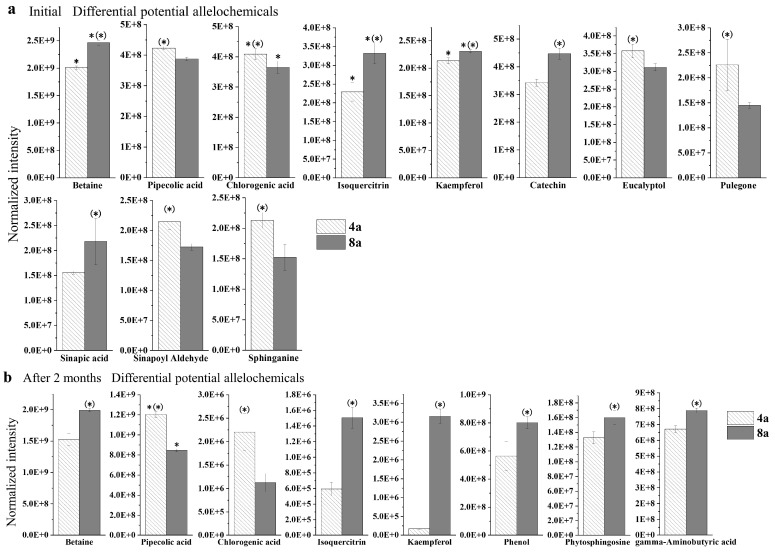
Normalization intensity of differential potential allelochemicals with contributive rate ≧ 2% in two-way ANOSIM and SIMPER analysis (Table 3) of 4- and 8-year-old *E. grandis* at the initial stage of leaf litter decomposition (**a**) and two months after decomposition (**b**). * indicates significant differences between the two decomposition times; (*) indicates significant differences between the two aged *E. grandis* stands; *p* < 0.05. Error bars indicate the standard errors.

The levels of some key differentially contributing metabolites in *E. grandis* litter during decomposition, such as betaine, chlorogenic acid, isoquercitrin, and kaempferol, generally decreased during decomposition. However, the pipecolic acid content increased after two months of decomposition. Catechin, eucalyptol, pulegone, sinapic acid, sinapoyl aldehyde, and sphinganine disappeared as the decomposition progressed, whereas phenol, phytosphingosine, and gamma-aminobutyric acid appeared as the decomposition process (Figure 4).

### 3.3. Effects of Leaf Litter Extract on Earthworms

#### 3.3.1. Survival Rate and Growth of *E. fetida*

Repeated ANOVA revealed that decomposition, exposure time, and dose significantly affected the survival and weight inhibition rates of *E. fetida* (Table 4). After 14 days of exposure to the 4-year-old litter treatment, the survival rate of *E. fetida* at C2 significantly increased after 10 days of decomposition and then remained stable over time (Table S1). The weight inhibition rate of *E. fetida* at C3 significantly increased after 20 days of decomposition and subsequently decreased over time. The weight inhibition rate of *E. fetida* decreased with increasing doses of litter extract under several treatments (Table S2).

#### 3.3.2. Physiological Properties of *E. fetida*

The content of total protein, MDA, and activities of SOD and AChE changed significantly with decomposition time, forest age, dose, and exposure time. Significant interactive effects on AChE activity were observed (Table 4). Under 4-year-old litter treatments, after seven days at C1 and C3 and 14 days of exposure at C1, C3, and C4 doses, the total protein content of *E. fetida* decreased significantly after 10–20 days of decomposition but remained stable or increased after 30 days and then decreased with time. Under 8-year-old litter treatments and seven days of exposure at C1 and C2, the total protein content of *E. fetida* decreased after 10 days of decomposition and then remained stable over time. After 14 days of exposure to C4, the protein content was initially stable but decreased after 45 days of decomposition (Figure S1).

After 7 or 14 days of exposure at C1 and C4 under the two mature litter treatments, the SOD activity of *E. fetida* fluctuated. It increased after 10–20 days of decomposition, decreased after 30 days, and then increased again after 45 days of decomposition. However, after seven days of exposure at C2 and C4, the SOD activity of *E. fetida* increased after 10 days of decomposition and then remained stable over time (Figure S2). After 14 days of exposure, the MDA content of *E. fetida* stimulated under 4-year-old litter treatments at C1, C2, and C4 increased after 20 days but decreased after 30 days of decomposition and then increased with time. For the treatments of the litter of 8-year-old trees, the MDA content at C3 after seven days of exposure was stimulated after ten days of decomposition and then stabilized over time. After 14 days of exposure at C1 and C2, the MDA content of *E. fetida* fluctuated and was stimulated after 20 or 45 days of decomposition (Figure S3).

After seven days of exposure, the AChE activity of *E. fetida* under 4-year-old litter treatments at C1 and C2 decreased after ten days of decomposition and then stabilized over time. After 14 days of exposure at C1 and C4, AChE activity decreased after 20–30 days, then stabilized with decomposition time. Under the treatment of 8-year-old litter at C2 after seven days of exposure, AChE activity decreased after 10 days and increased after 20 days of decomposition. However, after 14 days of exposure to C1, AChE activity was initially stable and then decreased after 30 or 45 days of decomposition (Figure S4).

The effects of dose and forest age on *E. fetida* were observed in several treatments. After 20 days of decomposition, the weight inhibitory rate of *E. fetida* increased with increasing doses of litter extracts of 4-year-old *E. grandis* and reached a maximum at C3. SOD activity of earthworms treated with 8-year-old litter increased at C1 and then stabilized with increasing doses. The total protein content of *E. fetida* was higher in the litter extracts of 4-year-old trees than in 8-year-old trees. However, SOD activity was higher in the litter of 8-year-old *E. grandis* than in 4-year-old *E. grandis* litter.

### 3.4. Effect of E. grandis Leaf Litter Extract on DNA Damage in E. fetida

Based on the above results, the toxic effects of *E. grandis* on *E. fetida* were stronger after 20–30 days of litter decomposition. We selected *E. grandis* litter after 30 days of decomposition to evaluate DNA damage in *E. fetida* using a comet assay. After 14 days of exposure, the litter extracts of 8-year-old *E. grandis* caused DNA damage to *E. fetida*, and no significant DNA damage to *E. fetida* was observed after treatment with litter extracts of 4-year-old trees. Compared with the control, the tail DNA content, tail moment, and OTM decreased significantly at C1 and then increased with increasing doses. At the highest concentration (C4), tail DNA content, tail length, and OTM were significantly higher in the litter of 8-year-old *E. grandis* than in the 4-year-old *E. grandis* litter (Table 5).

### 3.5. Correlation Analysis

**Figure 5:**
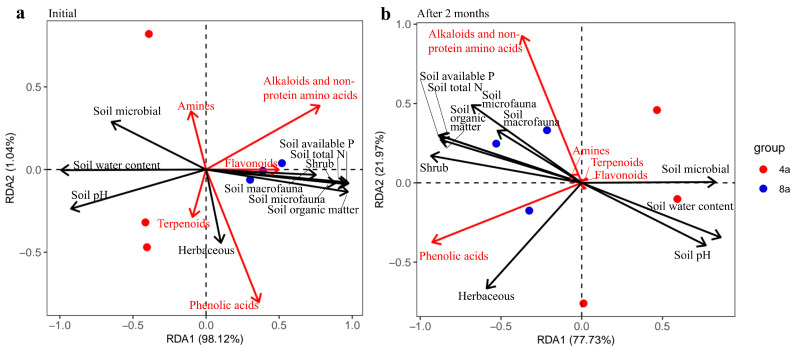
Redundancy analysis (RDA) diagram illustrating the relationship between soil physicochemical traits, soil biodiversity, and differential potential allelochemicals in 4- and 8-year-old *E. grandis* at the initial stage of leaf litter decomposition (**a**) and two months after decomposition (**b**).

Environmental changes between the two aged *E. grandis* affected the potential allelochemicals released by the litter during litter decomposition. The RDA showed that the first ordinate axes represented changes in most environmental factors, except for the diversity of herb species. At the initial decomposition stage, the second ordinate axes were closely correlated with the diversity of herb species. The contents of alkaloids, non-protein amino acids, and flavonoids in the initial litter were positively correlated with soil available P, total N, organic matter, shrub, and soil faunal diversity in the two-aged *E. grandis* plantations. The contents of terpenoids and phenolic acids were positively correlated, but that of amines was negatively correlated with changes in herb diversity. After two months of litter decomposition, the content of alkaloids and non-protein amino acids was positively correlated with changes in soil organic matter, available P, total N, soil faunal diversity, and shrub diversity. The phenolic acid content in *E. grandis* litter was positively correlated with changes in herb diversity. The amine, terpenoid, and flavonoid contents were not significantly correlated with the environmental factors (Figure 5, Table S3).

**Figure 6:**
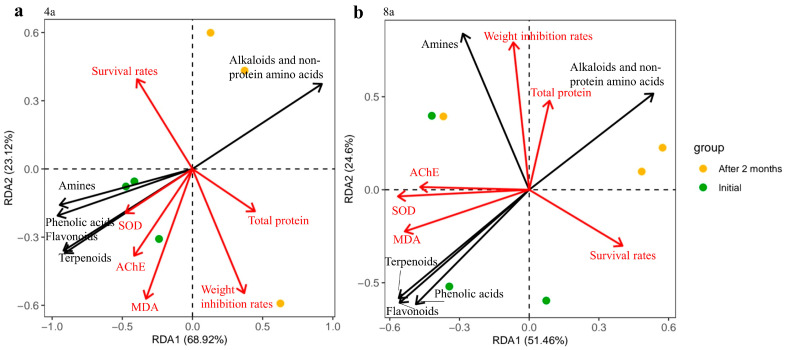
Redundancy analysis (RDA) diagram illustrating the relationship between differential potential allelochemicals and growth and physiological properties of *E. fetida* in the initial stage of leaf litter decomposition and two months after decomposition at 4-year-old (**a**) and 8-year-old *E. grandis* (**b**). The growth and physiological properties of *E. fetida* were standardized by Z-Score, in which the growth and physiological properties of *E. fetida* were 0 and 30 days of decomposition and 14 days of exposure to C4 concentration, respectively.

RDA also indicated that the responses of growth and physiological properties of *E. fetida* were correlated with the release of potential allelochemicals after two months of decomposition. For the 4-year-old stands, the first ordinate axis represents the changes in potential allelochemicals, whereas the second ordinate axis represents the changes in amines during litter decomposition in 8-year-old stands. Under the treatments of 4-year-old litter, the responses of SOD, AChE, and MDA in *E. fetida* were closely related to the changes in amines, phenolic acids, flavonoids, and terpenoids during litter decomposition. For the litter treatments of 8-year-old *E. grandis,* the changes in amines were responsible for the changes in the total protein and weight inhibitory rate of *E. fetida* (Figure 6, Table S4).

## 4. Discussion

Earthworms are epidermal respiratory soil macrofauna that can be easily absorbed into the body through respiration or can cause toxic effects through direct skin contact [51]. In this study, under most treatments, the survival rate of earthworms did not change significantly. The results indicated that allelochemicals in *E. grandis* litter were not maintained at sufficient concentrations, and earthworms activated the surface defense system in response to stimuli to avoid death [52]. However, inhaled and absorbed toxic chemicals inhibited this increase in earthworm weight. The weight inhibition rate of *E. fetida* increased significantly when treated with litter extracts after 20 days of decomposition. Earthworms reduce their food intake while avoiding the intake of toxic substances that are involved in the inhibitory effects on the weight of *E. fetida* [53].

Earthworms also adapt to environmental stress by adjusting their physiological activities. In this study, the total protein content and activities of SOD and AChE decreased, but the MDA content of *E. fetida* increased after 20 or 30 days of decomposition. The results indicated that the phytotoxicity of the litter extracts of *E. grandis* on *E. fetida* was severe at the initial stage of approximately 20–30 days of decomposition, followed by weaker toxicity as decomposition progressed. These results were consistent with previous studies, indicating that the most inhibitory effects occurred in the early stage of decomposition and then weakened in phytotoxicity with decomposition [11,33]. The initial decomposition phase of litter consists of comminution and release of the soluble fraction, followed by the degradation of cellulose and hemicellulose and retarded decomposition caused by inhibitory compounds such as tannins and lignin at the late stage of decomposition [26,54,55]. Several studies have reported a rapid loss of soluble phenolics from the *Eucalyptus* litter within the first week or month [33,36]. The rapid degradation of phenolics might be attributed to chemical processes after leaf fall, such as higher concentrations of oxidizing enzymes, particularly phenolases [34]. Subsequently, the rapid loss of phenolics from the *Eucalyptus* litter could be ascribed to leaching and sugar and microbial degradation [34,36,56]. A delay has been demonstrated between nutrient release from decomposing organic matter and root colonization of decomposing litter, which could be related to the occurrence of phytotoxic “windows” during the early stages of decomposition, which inhibits the development of the soil microbial community [11,57]. Despite a rapid decrease in phenolic compounds within a few days of leaf fall, Puig et al. (2018) observed that lettuce germination was completely inhibited at every sampling time [36], which demonstrated that *E. globulus* litter showed a continuous release of phenolic and volatile compounds during the 30 days of decomposition. As the decomposition period was prolonged, the phytotoxin levels declined when allelochemical inactivation was not balanced by new production. Previous studies have shown that there is a more resistant phenolic fraction after the initial rapid disappearance of phenolics and volatile compounds [36,58]. The relationship between allelochemicals and biological features, such as soil fauna, should refer to the resistant fraction that might be leached into the soil [11,34]. These soluble phenolics and volatiles entering the soil might be directly or indirectly toxic to soil fauna by changing the soil microbial community.

A total of 328 and 381 primary and secondary metabolites, respectively, were identified in the litter of the two mature *E. grandis*, and their presence, quantities, and composition varied significantly, depending on individual compounds as well as the decomposition process. Except for lipids in 4-year-old litter and amine in the litter of 8-year-old trees, which were stable, and the levels of nucleotides, alkaloids, and non-protein amino acids, which increased significantly, the levels of other primary and secondary metabolites reduced significantly after litter decomposition for two months. The secondary metabolites identified, such as catechin, eucalyptol, sinapic acid, and epicatechin, were partly in agreement with the allelochemicals previously identified in *Eucalyptus* litter [32,36,58]. In contrast, a few compounds, such as betaine, aminobutyric acid, and isoquercitrin, were not detected in previously identified *E. grandis* litter. This can be explained by the fact that allelochemicals are subjected to various biotic and abiotic processes, reduce their concentrations, and induce chemical transformation into other compounds [59,60]. The quantities of some of the main differentially contributing metabolites in *E. grandis* litter, such as betaine, chlorogenic acid, isoquercitrin, and kaempferol, generally decrease during decomposition. However, the quantity of pipecolic acid increased after two months of decomposition. Catechin, eucalyptol, pulegone, sinapic acid, sinapoyl aldehyde, and sphinganine disappeared as the decomposition proceeded, whereas phenol, phytosphingosine, and gamma-aminobutyric acid appeared as the decomposition proceeded. Although metabolites from *E. grandis* litter have not been consistently identified at each specific time point of decomposition, we also observed that the dynamics of allelochemical release were closely aligned with the phytotoxicity of *E. grandis* litter throughout the decomposition process. The main allelochemicals, including amines, phenolic acids, flavonoids, and terpenoids, explain the variation in the phytotoxicity of *E. fetida* during decomposition. Despite the insolubility of volatile organic compounds, such as terpenoids, their lower solubility and retention in soil particles have also been reported, which partly explains the phytotoxicity of eucalyptus litter on *E. fetida* [14,36].

Biochemical responses of earthworms have been regarded as a warning system for environmental stress [61]. Proteins are important components of an organism and regulate various physio-metabolic processes [62]. In this study, the total protein content of *E. fetida* decreased significantly after 20 days of litter decomposition. After the initial fragmentation of the litter, the rapid release of potential allelochemicals promoted the secretion of protein enzymes from the cell wall and intestinal tract and altered membrane permeability, consuming total protein from *E. fetida* [63,64]. Subsequent release of the soluble C fraction and nutrients gradually reduced the stimulation of earthworms, provided food resources, and increased total protein [20]. SOD is an important enzyme in the antioxidant system that prevents lipid peroxidation [65]. The SOD activity of *E. fetida* was initially increased but decreased after 30 days of decomposition and then increased. The increase in SOD activity implies that earthworms suffered from oxidative stress, as reflected by the production of superoxide anion radicals to reduce the cellular stress response [66,67]. The MDA content was also stimulated after 20 days of decomposition and then stabilized or decreased with decomposition time. MDA is the final product of lipid peroxidation, caused by an increase in oxygen free radicals, reflecting the degree of lipid peroxidation and damage to the organism [68,69]. The AChE is a specific filament amino acid hydrolase that promotes neuronal development and regeneration [70]. AChE activity of *E. fetida* was initially stable but decreased after 30 days of decomposition and then stabilized or increased with decomposition. Inhibition of AChE activity and behavioral alterations stimulate cholinergic receptors, ultimately leading to uncoordinated movements and neuromuscular paralysis [71,72]. In this study, a lack of avoidance response was observed after treatment with litter after 30 days of decomposition. Therefore, leaf litter of *E. grandis* after decomposition for 20–30 days had a stronger effect on the physiological properties of *E. fetida.*

In addition, the dynamics of allelochemicals and phytotoxicity of *E. grandis* litter on *E. fetida* varied considerably depending on plantation forest age. In this study, the total protein content of *E. fetida* was higher in the litter of 4-year-old trees than in 8-year-old trees, whereas the SOD activity of earthworms showed the opposite trend. Natural antioxidant defenses can be overwhelmed when enzymes cannot scavenge free radicals from pollutants, causing serious cellular DNA damage [73,74]. Comet assays have been used to detect DNA damage in *E. fetida* exposed to *E. grandis* litter extract. Olive tail moment (OTM) is the most sensitive comet parameter, which reveals the severity and quantity of DNA damage [75,76]. In this study, the increment of tail DNA, comet tail length, and OTM indicated that litter extracts of 8-year-old *E. grandis* caused more DNA damage to *E. fetida* than 4-year-old trees. DNA damage in earthworms leads to compromised immunity, rendering *E. fetida* vulnerable [74]. In addition, the litter extracts of *E. grandis* showed more severe toxic effects on *E. fetida* with increasing doses [77]; however, DNA damage decreased at the lowest dose. Lower concentrations of allelochemicals can weaken phytotoxicity and enhance the growth and survival of the earthworm.

Previous studies at the study sites indicated that 4-year-old and 8-year-old trees were the critical turning points for the increment of potential allelochemicals in litter across a range of *E. grandis* plantations. In the present study, except for the higher amount of amino acids and peptides, terpenoids, and amines in the initial litter and lipids after 2-month decomposition, the other primary and secondary metabolites were in greater abundance in the litter of 8-year-old *E. grandis* than in 4-year-old *E. grandis* litter. In general, mature leaves produce more secondary metabolic substances than young leaves, whereas young leaves have more abundant amino and organic acids [78]. Given the higher growth rate of *E. grandis* at four years, intra- and inter-specific competition for soil resources was severe, and *E. grandis* would invest more resources in chemical defense mechanisms [7,39]. However, the rapid growth rate of the tree created a relatively open canopy before the rotation period (approximately four years), which facilitated the development of soil microbial communities and, to some extent, might promote the degradation of the accumulated production of allelochemicals. Previous studies at the study sites also observed a slight difference in the diversity of the soil microbial community between the two-aged stands; however, they were higher in 4-year-old stands than in 8-year-old stands [79]. External environmental factors could also explain the between-age differences in potential allelochemicals [12,80]. Soluble compounds may have been efficiently leached out of young *E. grandis* litter under the relatively open canopy of 4-year-old stands. The closed canopy and developed understory plant structure and diversity in 8-year-old *E. grandis* stands increased the interception of rainfall, and phenolics were less efficiently leached out and consequently persisted longer in the mature leaf litter. As the decomposition proceeded, more abundant terpenoids were released quickly in the young leaf litter but retained for longer periods in mature *E. grandis* litter [14,81]. In addition, pH can affect the solubility of allelochemicals with increasing toxicity under acidic conditions in 8-year-old stands [11,82]. All of these factors explain the severe phytotoxicity of litter extracts of 8-year-old *E. grandis* compared to 4-year-old *E. grandis*.

## 5. Conclusions

In conclusion, this study demonstrated the occurrence of litter phytotoxicity in *E. fetida* with clear, dynamic patterns during the initial decomposition stage. The litter of *E. grandis,* after decomposition for 20–30 days, exerted stronger phytotoxicity on the growth and biochemical characteristics of *E. fetida*, and the toxicity was weakened by decomposition. *E. fetida* individuals exhibited significant inhibition of weight and the activities of AChE under litter treatment after 20–30 days of decomposition, whereas the activities and MDA content were stimulated at this stage. The phytotoxicity dynamics of decaying litter on *E. fetida* might be involved in the resistance of phenolic and volatile fractions after the initial fast disappearance of allelochemicals. Compared with the litter of 4-year-old trees, *E. fetida* exhibited a more severe phytotoxicity response to protein content, stimulation of SOD activity, and DNA damage in the litter extracts of 8-year-old *E. grandis*. Consistently, an increased abundance of potential allelochemicals was observed in the litter of 8-year-old *E. grandis*. However, the expression of allelopathy in 8-year-old litter does not necessarily need to outcompete 4-year-old litter under natural conditions because of the developed stand structure, environmental heterogeneity, and abundant precipitation in this region. Overall, our results suggest that the use of scientific management measures, such as the appropriate removal of leaf litter in the early stages of decomposition, may help support greater biodiversity in *E. grandis* plantations.

## Figures and Tables

**Table 1:** Soil characteristics of two differently aged *E. grandis* plantations.

Stand	Soil Bulk Density	Soil pH	Soil Water Content	Soil Organic Matter	Soil Total N	Soil Available *p*
4a	1.33 ± 0.08	3.99 ± 0.03 *	29.38 ± 0.01 ***	15.90 ± 0.88	0.27 ± 0.03	2.54 ± 0.24
8a	1.22 ± 0.12	3.90 ± 0.03	28.99 ± 0.02	24.56 ± 0.58 ***	0.91 ± 0.06 ***	6.89 ± 0.26 ***

* indicates significant differences between soil properties depending on forest ages. *, *p* < 0.05; ***, *p* < 0.001; mean ± standard error (SE).

**Table 2:** Plant, soil microbial, and soil fauna diversity in the two different aged *E. grandis* plantations.

		Species Number	Density (Ind m^−2^)	Shannon– Wiener	Pielou	Berger–Parker
Plant’s diversity	Shrub (4a)	3.00 ± 0.87	0.51 ± 0.18	0.75 ± 0.14	0.76 ± 0.08	0.68 ± 0.03 ***
	Shrub (8a)	6.50 ± 0.43 **	1.11 ± 0.18 *	1.65 ± 0.05 ***	0.82 ± 0.02	0.35 ± 0.00
	Herbaceous (4a)	7.50 ± 2.18	48.00 ± 18.99	1.57 ± 0.21	0.73 ± 0.06	0.40 ± 0.03
	Herbaceous (8a)	7.75 ± 1.30	54.25 ± 4.63	1.64 ± 0.20	0.79 ± 0.07	0.39 ± 0.03
Soil faunal diversity	Macrofauna (4a)	6.42 ± 0.14	54.00 ± 11.27	1.58 ± 0.09	0.80 ± 0.05	0.40 ± 0.05 *
	Macrofauna (8a)	7.50 ± 1.30	62.33 ± 19.73	1.79 ± 0.09 *	0.86 ± 0.07	0.31 ± 0.02
	Microfauna (4a)	9.08 ± 0.95	8441.67 ± 1203.21	1.66 ± 0.02	0.64 ± 0.07	0.40 ± 0.02
	Microfauna (8a)	10.67 ± 0.14	12141.67 ± 2546.61	1.79 ± 0.07 *	0.59 ± 0.05	0.37 ± 0.04
		OTUs	Shannon–Wiener	Simpson	ACE	Chao1
Soil microbial diversity	(4a)	1623.08 ± 20.12	5.94 ± 0.07	0.99 ± 0.00	2474.72 ± 35.69	2312.38 ± 16.72
	(8a)	1537.58 ± 91.82	5.72 ± 0.17	0.98 ± 0.01	2398.02 ± 127.48	2208.14 ± 145.15

* indicates significant differences between soil properties depending on forest ages. *, *p* < 0.05; **, *p* < 0.01; ***, *p* < 0.001; mean ± standard error (SE).

**Table 3:** Results of analysis of similarity (ANOSIM) for differential potential allelochemicals of *E. grandis* between forest ages and decomposition times and analysis of similarity percentage (SIMPER).

Effects	R and Significance	Main Contributive Compounds for Variation
**Two-way ANOSIM**		
Forest age	(1) ***	Betaine, pipecolic acid, phenol, gamma-aminobutyric acid, catechin, chlorogenic acid, eucalyptol, isoquercitrin, kaempferol, pulegone, sinapic acid, sinapoyl aldehyde, and sphinganine
Decomposition time	(1) ***	Gamma-aminobutyric acid, phenol, pipecolic acid, betaine, catechin, chlorogenic acid, eucalyptol, isoquercitrin, kaempferol, sinapoyl aldehyde, pulegone, sinapic acid, sphinganine, and phytosphingosine
**One-way ANOSIM of forest age effects**		
Initial	(1) *	Betaine, methyleugenol, catechin, isoquercitrin, pulegone, sinapic acid, Sphinganine, 4-hydroxycinnamic acid, eucalyptol, Taraxerol, chlorogenic acid, sinapoyl aldehyde, and pipecolic acid
After 2 months	(1) *	Betaine, pipecolic acid, phenol, gamma-aminobutyric acid, and saccharopine
**One-way ANOSIM of decomposition time effects**		
4 years	(1) *	Betaine, 2-aminophenol, Taraxerol, gamma-aminobutyric acid, phenol, pipecolic acid, chlorogenic acid, eucalyptol, catechin, isoquercitrin, and pulegone
8 years	(1) *	Betaine, 2-aminophenol, phenol, gamma-aminobutyric acid, Taraxerol, catechin, pipecolic acid, chlorogenic acid, isoquercitrin, and eucalyptol

Forest ages: 4 and 8 years old. Data in parentheses are the R value. Significant effects are indicated in superscript: *, *p* < 0.05; ***, *p* < 0.001. The rightmost column is the contributive compounds (SIMPER analysis, contributive rate ≧ 2%) to the variation in differential potential allelochemicals composition among study sites or decomposition times.

**Table 4:** Repeated measures ANOVA of the effects of forest age, decomposition time, exposure time, and dose on growth and physiological characteristics of *E. fetida*.

	Survival Rate		Weight Inhibition Rate		Total Protein Content		SOD Activity		MDA Content		AChE Activity	
	*F*	*p*	*F*	*p*	*F*	*p*	*F*	*p*	*F*	*p*	*F*	*p*
Decomposition time	17.476	0.000 ***	5.498	0.000 ***	41.199	0.000 ***	69.155	0.000 ***	29.708	0.000 ***	43.625	0.000 ***
Age	0.129	0.720	0.088	0.767	5.130	0.026 *	10.673	0.001 ***	2.596	0.110	2.815	0.097
Dose	0.587	0.673	4.680	0.002 **	3.034	0.021 *	7.769	0.000 ***	2.708	0.034 *	6.349	0.000 ***
Exposure time	134.20	0.000 ***	420.00	0.000 ***	22.767	0.000 ***	2.395	0.125	94.713	0.000 ***	8.834	0.004 **
Age × Dose	1.863	0.123	2.372	0.057	0.816	0.518	2.345	0.060	0.532	0.712	1.100	0.361
Age × Decomposition time	0.662	0.620	2.455	0.051	0.905	0.464	0.865	0.488	1.065	0.378	3.667	0.008 **

* indicates significant differences. *, *p* < 0.05; **, *p* < 0.01; ***, *p* < 0.001.

**Table 5:** DNA damage effects of *E. grandis* litter extracts from different aged stands evaluated via a comet assay on coelomocytes from *E. fetida*.

Dose	Tail DNA (%)		Tail Moment		Olive Tail Moment		Tail Length	
	4a	8a	4a	8a	4a	8a	4a	8a
CK	1.51 ± 0.81	1.51 ± 0.81 (ab)	0.19 ± 0.12	0.19 ± 0.12 (ab)	0.46 ± 0.21	0.46 ± 0.21 (ab)	7.46 ± 2.31	7.46 ± 2.31
C1	1.63 ± 0.48	1.17 ± 0.29 (b)	0.34 ± 0.2	0.12 ± 0.03 (b)	0.50 ± 0.17	0.29 ± 0.07 (b)	6.28 ± 0.69	5.02 ± 0.49
C2	2.16 ± 0.87	1.52 ± 0.13 (ab)	0.52 ± 0.32	0.52 ± 0.18 (ab)	0.75 ± 0.33	0.56 ± 0.04 (ab)	9.04 ± 4.48	7.98 ± 1.44
C3	1.50 ± 0.54	2.84 ± 1.32 (ab)	0.24 ± 0.1	0.79 ± 0.56 (a)	0.46 ± 0.21	0.97 ± 0.55 (a)	6.85 ± 2.02	11.75 ± 6.19
C4	1.03 ± 0.24	3.20 ± 0.74 (a)(*)	0.12 ± 0.07	0.82 ± 0.58 (a)	0.27 ± 0.08	1.04 ± 0.27 (a)(*)	5.33 ± 1.34	11.70 ± 3.28 (*)

Lowercase letters in parentheses indicate significant differences among various concentrations; (*) indicates significant differences between two aged *E. grandis* stands; *p* < 0.05; mean ± standard error (SE).

## Data Availability

Data will be made available upon request.

## Supplementary Materials

The following supporting information can be downloaded at https://www.mdpi.com/article/10.3390/plants13172415/s1. Table S1: Effects of litter extracts from two aged *E. grandis* stands during decomposition on the survival rate of *E. fetida* after 7 and 14 days of exposure; Table S2: Effects of litter extracts from two aged E. grandis stands during decomposition on the weight inhibition rate of *E. fetida* after 7 and 14 days of exposure; Table S3: Correlations between the first two axes and environmental factors in RDA and the determination coefficient (r^2^) and significance test (Pr) of the correlation between environmental factors and differential potential allelochemicals using the envfit function (R package vegan); Table S4: Correlations between the first two axes and differential potential allelochemicals in RDA, and the determination coefficient (r^2^) and significance test (Pr) of the correlation between differential potential allelochemicals and growth and physiological properties of *E. fetida* using the envfit function (R package vegan); Figure S1: Effects of litter extracts from two mature E. grandis stands during decomposition on the total protein content of *E. fetida* after 7 and 14 days of exposure; Figure S2: Effects of litter extracts from two mature *E. grandis* stands during decomposition on SOD activity of *E. fetida* after 7 and 14 days of exposure; Figure S3: Effects of litter extracts from two mature *E. grandis* stands during decomposition on MDA content of *E. fetida* after 7 and 14 days of exposure; Figure S4: Effects of litter extracts from two mature *E. grandis* stands during decomposition on AChE activity of *E. fetida* after 7 and 14 days of exposure.

## References

[1] Zhang D.J., Li J.J., Huang Y.M., Gao S., Zhang J. (2022). Root-soil facilitation in mixed *Eucalyptus grandis* plantations including nitrogen-fixing species. For. Ecol. Manag..

[2] Massuque J., Roque Lima M.D., Müller Da Silva P.H., de Paula Protásio T., Trugilho P.F. (2023). Potential of charcoal from non-commercial *Corymbia* and *Eucalyptus* wood for use in the steel industry. Renew. Energy.

[3] National Forestry and Grassland Administration (2020). China Forest Resource Report.

[4] Forrester D.I., Bauhus J., Cowie A.L., Vanclay J.K. (2006). Mixed-species plantations of *Eucalyptus* with nitrogen-fixing trees: A review. For. Ecol. Manag..

[5] Rodríguez-Loinaz G., Amezaga I., Onaindia M. (2013). Use of native species to improve carbon sequestration and contribute towards solving the environmental problems of the timberlands in Biscay, northern Spain. J. Environ. Manag..

[6] Ahmed R., Hoque A.T.M.R., Hossain M.K. (2008). Allelopathic effects of leaf litters of *Eucalyptus camaldulensis* on some forest and agricultural crops. J. For. Res..

[7] Zhang D.J., Zhang J., Yang W.Q., Wu F.Z. (2010). Potential allelopathic effect of *Eucalyptus grandis* across a range of plantation ages. Ecol. Res..

[8] Chu C.J., Mortimer P.E., Wang H.C., Wang Y.F., Liu X.B., Yu S.X. (2014). Allelopathic effects of *Eucalyptus* on native and introduced tree species. For. Ecol. Manag..

[9] Rice E.L. (1984). Allelopathy.

[10] Inderjit, Callaway R.M. (2003). Experimental designs for the study of allelopathy. Plant Soil.

[11] Bonanomi G., Sicurezza M.G., Caporaso S., Esposito A., Mazzoleni S. (2006). Phytotoxicity dynamics of decaying plant materials. New Phytol..

[12] Chomel M., Fernandez C., Bousquet-Mélou A., Gers C., Monnier Y., Santonja M., Gauquelin T., Gros R., Lecareux C., Baldy V. (2014). Secondary metabolites of *Pinus halepensis* alter decomposer organisms and litter decomposition during afforestation of abandoned agricultural zones. J. Ecol..

[13] Kruidhof H.M., Gallandt E.R., Haramoto E.R., Bastiaans L. (2011). Selective weed suppression by cover crop residues: Effects of seed mass and timing of species’ sensitivity. Weed Res..

[14] Tang Z.Q., Zhang J., Yu J.L., Wang C.Z., Zhang D.J. (2017). Allelopathic effects of volatile organic compounds from *Eucalyptus grandis* rhizosphere soil on *Eisenia fetida* assessed using avoidance bioassays, enzyme activity, and comet assays. Chemosphere.

[15] Molina A., Reigosa M.J., Carballeira A. (1991). Release of allelochemical agents from litter, throughfall, and topsoil in plantations of *Eucalyptus globulus* Labill in Spain. J. Chem. Ecol..

[16] Fang B.Z., Yu S.X., Wang Y.F., Qiu X., Cai C.X., Liu S.P. (2009). Allelopathic effects of *Eucalyptus urophylla* on ten tree species in south China. Agrofor. Syst..

[17] Mohamed E.I., Gad E.K. (2019). *Eucalyptus citriodora* leaf extract as a source of allelochemicals for weed control in pea fields compared with some chemical herbicides. J. Plant Prot. Res..

[18] Inderjit, Wardle D.A., Karban R., Callaway R.M. (2011). The ecosystem and evolutionary contexts of allelopathy. Trends Ecol. Evol..

[19] Boeno D., Silva R.F., Almeida H.S., Rodrigues A.C., Vanzan M., Andreazza R. (2020). Influence of eucalyptus development under soil fauna. Braz. J. Biol..

[20] Rodriguez-Campos J., Dendooven L., Alvarez-Bernal D., Contreras-Ramos S.M. (2014). Potential of earthworms to accelerate removal of organic contaminants from soil: A review. Appl. Soil Ecol..

[21] Zhang C., Dai J., Chen X.F., Li H.H., Lavelle P. (2020). Effects of a native earthworm species (*Amynthas morrisi*) and *Eisenia fetida* on metal fractions in a multi-metal polluted soil from South China. Acta Oecologica.

[22] Xie X.C., Qian Y., Wu Y.X., Yin J., Zhai J.P. (2013). Effects of decabromodiphenyl ether (BDE-209) on the avoidance response, survival, growth and reproduction of earthworms (*Eisenia fetida*). Ecotoxicol. Environ. Saf..

[23] Markert B., Breure A.M., Zechmeister H.G. (2003). Bioindicators and Biomonitors: Principles, Concepts and Applications.

[24] Gomez-Eyles J.L., Svendsen C., Lister L., Martin H., Hodson M.E., Spurgeon D.J. (2009). Measuring and modelling mixture toxicity of imidacloprid and thiacloprid on *Caenorhabditis elegans* and *Eisenia fetida*. Ecotoxicol. Environ. Saf..

[25] Makkonen M., Berg M.P., van Logtestijn R.S.P., van Hal J.R., Aerts R. (2013). Do physical plant litter traits explain non-additivity in litter mixtures? A test of the improved microenvironmental conditions theory. Oikos.

[26] Gartner T.B., Cardon Z.G. (2004). Decomposition Dynamics in Mixed-Species Leaf Litter. Oikos.

[27] Hättenschwiler S., Tiunov A.V., Scheu S. (2005). Biodiversity and Litter Decomposition in Terrestrial Ecosystems. Annu. Rev. Ecol. Evol. Syst..

[28] Wang L.F., Zhou Y., Chen Y.M., Xu Z.F., Zhang J., Liu Y. (2023). Home-field advantage and ability alter labile and recalcitrant litter carbon decomposition in an alpine forest ecotone. Plant Soil.

[29] Hättenschwiler S., Vitousek P.M. (2000). The role of polyphenols in terrestrial ecosystem nutrient cycling. Trends Ecol. Evol..

[30] Coq S., Souquet J., Meudec E., Cheynier V., Hättenschwiler S. (2010). Interspecific variation in leaf litter tannins drives decomposition in a tropical rain forest of French Guiana. Ecology.

[31] Zhang Y., Li X., Zhang D.J., Qin Y., Zhou Y., Song S.M., Zhang J. (2020). Characteristics of fungal community structure during the decomposition of mixed foliage litter from *Pinus massoniana* and broadleaved tree species in southwestern China. J. Plant Ecol..

[32] Li J.J., Huang Y.M., Chen L.H., Gao S., Zhang J., Zhang D.J. (2023). Understory plant diversity and phenolic allelochemicals across a range of *Eucalyptus grandis* plantation ages. J. For. Res..

[33] An M., Pratley J.E., Haig T. (2001). Phytotoxicity of Vulpia Residues: IV. Dynamics of Allelochemicals During Decomposition of Vulpia Residues and Their Corresponding Phytotoxicity. J. Chem. Ecol..

[34] Bernhard-Reversat F., Main G., Holl K., Loumeto J., Ngao J. (2003). Fast disappearance of the water-soluble phenolic fraction in eucalypt leaf litter during laboratory and field experiments. Appl. Soil Ecol..

[35] Blum U. (2014). Plant-Plant Allelopathic Interactions Ⅱ: Laboratory Bioassays for Water-Soluble Compounds with an Emphasis on Phenolic Acids.

[36] Puig C.G., Gonçalves R.F., Valentão P., Andrade P.B., Reigosa M.J., Pedrol N. (2018). The Consistency between Phytotoxic Effects and the Dynamics of Allelochemicals Release from *Eucalyptus globulus* Leaves Used as Bioherbicide Green Manure. J. Chem. Ecol..

[37] Liu S., Qin F.C., Yu S.X. (2018). *Eucalyptus urophylla* root-associated fungi can counteract the negative influence of phenolic acid allelochemicals. Appl. Soil Ecol..

[38] Zhang D.J., Zhang J., Yang W.Q., Wu F.Z. (2012). Effects of afforestation with *Eucalyptus grandis* on soil physicochemical and microbiological properties. Soil Res..

[39] Zhang D.J., Zhang J., Yang W.Q., Wu F.Z., Huang Y.M. (2014). Plant and soil seed bank diversity across a range of ages of *Eucalyptus grandis* plantations afforested on arable lands. Plant Soil.

[40] Zhang D.J., Zhang J., Yang W.Q., Wu F.Z., Huang Y.M., Zhang Z.W., Wang X., Wang X.Q., Zhu L. (2013). Plant’s and soil organism’s diversity across a range of *Eucalyptus grandis* plantation ages. Acta Ecol. Sin..

[41] Research Group of Chinese Soil Taxonomy (2001). Keys to Chinese Soil Taxonomy.

[42] OECD (2004). Guideline for Testing of Chemicals. No.222. Earthworm Reproduction Test (Eisenia fetida/andrei).

[43] (2008). Soil Quality: Avoidance Test for Testing the Quality of Soils and Effects of Chemicals on Behavior—Part 1: Test with Earthworms (*Eisenia fetida* and *Eisenia andrei*).

[44] Yu J.L., Zhang J., Tang Z.Q., Wang C.Z., Qian L.Z., Zhang D.J. (2017). Variations of growth and physiological indicators of *Eisenia foetida* in soils across a chronosequence of *Eucalyptus grandis* plantations. Chin. J. Ecol..

[45] Smith P.K., Krohn R.I., Hermanson G.T., Mallia A.K., Gartner F.H., Provenzano M.D., Fujimoto E.K., Goeke N.M., Olson B.J., Klenk D.C. (1985). Measurement of protein using bicinchoninic acid. Anal. Biochem..

[46] Cortés-Ríos J., Zárate A.M., Figueroa J.D., Medina J., Fuentes-Lemus E., Rodríguez-Fernández M., Aliaga M., López-Alarcón C. (2020). Protein quantification by bicinchoninic acid (BCA) assay follows complex kinetics and can be performed at short incubation times. Anal. Biochem..

[47] Ōyanagui Y. (1984). Reevaluation of assay methods and establishment of kit for superoxide dismutase activity. Anal. Biochem..

[48] Kosugi H., Kikugawa K. (1985). Thiobarbituric acid reaction of aldehydes and oxidized lipids in glacial acetic acid. Lipids.

[49] Gorun V., Proinov I., Băltescu V., Balaban G., Bârzu O. (1978). Modified Ellman procedure for assay of cholinesterases in crude enzymatic preparations. Anal. Biochem..

[50] Eyambe G.S., Goven A.J., Fitzpatrick L.C., Venables B.J., Cooper E.L. (1991). A non-invasive technique for sequential collection of earthworm (*Lumbricus terrestris*) leukocytes during subchronic immunotoxicity studies. Lab. Anim..

[51] Ribeiro S., Sousa J.P., Nogueira A.J.A., Soares A.M.V.M. (2001). Effect of Endosulfan and Parathion on Energy Reserves and Physiological Parameters of the Terrestrial Isopod *Porcellio dilatatus*. Ecotoxicol. Environ. Saf..

[52] Verdú I., Trigo D., Martínez-Guitarte J.L., Novo M. (2018). Bisphenol A in artificial soil: Effects on growth, reproduction and immunity in earthworms. Chemosphere.

[53] Olvera-Velona A., Capowiez Y., Mascle O., Ortiz-Hernandez L., Benoit P. (2008). Assessment of the toxicity of ethyl-parathion to earthworms (*Aporrectodea caliginosa*) using behavioural, physiological and biochemical markers. Appl. Soil Ecol..

[54] Lummer D., Scheu S., Butenschoen O. (2012). Connecting litter quality, microbial community and nitrogen transfer mechanisms in decomposing litter mixtures. Oikos.

[55] Wu F.Z., Peng C.H., Yang W.Q., Zhang J., Han Y., Mao T. (2014). Admixture of alder (*Alnus formosana*) litter can improve the decomposition of eucalyptus (*Eucalyptus grandis*) litter. Soil Biol. Biochem..

[56] Maity S.K., Joy V.C. (1999). Impact of antinutritional chemical compounds of leaf litter on detritivore soil arthropod fauna. J. Ecobiol..

[57] Hodge A., Stewart J., Robinson D., Griffiths B.S., Fitter A.H. (1998). Root proliferation, soil fauna and plant nitrogen capture from nutrient-rich patches in soil. New Phytol..

[58] Reigosa M.J., Pazos-Malvido E. (2007). Phytotoxic Effects of 21 Plant Secondary Metabolites on *Arabidopsis thaliana* Germination and Root Growth. J. Chem. Ecol..

[59] Otte B.A., Rice C.P., Davis B.W., Schomberg H.H., Mirsky S.B., Tully K.L. (2020). Phenolic acids released to soil during cereal rye cover crop decomposition. Chemoecology.

[60] Bonanomi G., Zotti M., Idbella M., Mazzoleni S., Abd-ElGawad A.M. (2021). Microbiota modulation of allelopathy depends on litter chemistry: Mitigation or exacerbation?. Sci. Total Environ..

[61] Gu H.T., Yuan Y.D., Cai M., Wang D.S., Lv W.G. (2021). Toxicity of isoprocarb to earthworms (*Eisenia fetida*): Oxidative stress, neurotoxicity, biochemical responses and detoxification mechanisms. Environ. Pollut..

[62] Chen L.Y., Bai J., Wan J., Song Y., Xiang G.H., Duan R.Y., Zheng Y. (2024). Endocrine system, cell growth and death, and energy metabolism induced by Sb(III) exposure in earthworm (*Pheretima guillemi*) revealed by transcriptome and metabolome analysis. Environ. Pollut..

[63] Zhao H.B., Shi S.Y., Zhao H., Guo J., Yang Z., Gao H.S., Lu F.P. (2019). Proteomic analysis of the earthworm *Eisenia fetida* exposed to oxytetracycline in soil. RSC Adv..

[64] Rong H., Wang C.R., Liu H.T., Zhang M., Yuan Y.T., Pu Y.J., Huang J., Yu J.Y. (2020). Biochemical Toxicity and Potential Detoxification Mechanisms in Earthworms *Eisenia fetida* Exposed to Sulfamethazine and Copper. Bull. Environ. Contam. Toxicol..

[65] Yao X.F., Liang C.L., Lv H.J., Liu W.R., Wang Q., Ding J., Li X.X., Wang J. (2024). Expanding the insight of ecological risk on the novel chiral pesticide mefentrifluconazole: Mechanism of enantioselective toxicity to earthworms (*Eisenia fetida*). J. Hazard. Mater..

[66] Shao Y.T., Wang J.H., Wang J., Du Z.K., Li B., Zhu L.S., Juhasz A., Liu X.Y., Xu Y.Q., Li W.X. (2019). Oxidative stress and genotoxic effects in earthworms induced by five imidazolium bromide ionic liquids with different alkyl chains. Chemosphere.

[67] Song P.P., Gao J.P., Li X.X., Zhang C., Zhu L.S., Wang J.H., Wang J. (2019). Phthalate induced oxidative stress and DNA damage in earthworms (*Eisenia fetida*). Environ. Int..

[68] Shi Z.M., Yan J.H., Ren X.N., Wen M., Zhao Y.H., Wang C.Y. (2021). Effects of biochar and thermally treated biochar on *Eisenia fetida* survival, growth, lysosomal membrane stability and oxidative stress. Sci. Total Environ..

[69] Bao X., Wang Z.J., Liu L., Wang D.W., Gu Y.T., Chen L., Chen X.J., Meng Z.Y. (2024). The combined effects of azoxystrobin and different aged polyethylene microplastics on earthworms (*Eisenia fetida*): A systematic evaluation based on oxidative damage and intestinal function. Sci. Total Environ..

[70] Gautam K., Dwivedi S., Verma R., Vamadevan B., Patnaik S., Anbumani S. (2024). Combined effects of polyethylene microplastics and carbendazim on *Eisenia fetida*: A comprehensive ecotoxicological study. Environ. Pollut..

[71] He F.L., Liu R.T. (2023). Mechanistic insights into phenanthrene-triggered oxidative stress-associated neurotoxicity, genotoxicity, and behavioral disturbances toward the brandling worm (*Eisenia fetida*) brain: The need for an ecotoxicological evaluation. J. Hazard. Mater..

[72] Ali H.M., Abdel-Aty B., El-Sayed W., Mariy F.M., Hegazy G.M., Mohamed R.A., Zoghly H.M. (2024). Imidacloprid effects on acetylcholinesterase and nicotinic acetylcholine receptor in *Apis mellifera*. Experimental and molecular modeling approaches. Chemosphere.

[73] Zhao S.L., Wang Y.L., Duo L. (2021). Biochemical toxicity, lysosomal membrane stability and DNA damage induced by graphene oxide in earthworms. Environ. Pollut..

[74] Li X.X., Jiang N., Zhang J., Yao X.F., Liu W.R., Wang Q., Ding J., Hu Z.R., Zhu L.S., Wang J.H. (2024). Soil health hazards of di(2-ethylhexyl) phthalate: New perspectives on earthworms from different ecological niches DNA damage, gut microbial disruption and soil enzyme changes. J. Hazard. Mater..

[75] Espinosa-Reyes G., Costilla-Salazar R., Pérez-Vázquez F.J., González-Mille D.J., Flores-Ramírez R., Del Carmen Cuevas-Díaz M., Medellin-Garibay S.E., Ilizaliturri-Hernández C.A. (2019). DNA damage in earthworms by exposure of Persistent Organic Pollutants in low basin of Coatzacoalcos River, Mexico. Sci. Total Environ..

[76] Yan X.J., Wang J.H., Zhu L.S., Wang J., Li S.Y., Kim Y.M. (2021). Oxidative stress, growth inhibition, and DNA damage in earthworms induced by the combined pollution of typical neonicotinoid insecticides and heavy metals. Sci. Total Environ..

[77] Xue Y.N., Li Z.G., Liu C., Liu D.M., Wang J.H., Liu C., Xia X.M. (2023). Effect of different exposure times and doses of cyantraniliprole on oxidative stress and genotoxicity in earthworms (*Eisenia fetida*). Chemosphere.

[78] Lin W.D., Li J.M. (2021). Comparative Analysis of the Main Metabolites in Tender and Matured Leaves of *Cyclocarya Paliurus* Based on Metabolomic Data. J. Taizhou Univ..

[79] Wang C.Z., Zhang D.J., Yu J.L., Tang Z.Q., Zhang J. (2019). Soil Microbial Community Diversity and Composition across a Range of *Eucalyptus grandis* Plantations of Different Ages. Int. J. Agric. Biol..

[80] Kuiters A.T., Sarink H.M. (1986). Leaching of phenolic compounds from leaf and needle litter of several deciduous and coniferous trees. Soil Biol. Biochem..

[81] Ormeño E., Fernandez C., Mévy J. (2007). Plant coexistence alters terpene emission and content of Mediterranean species. Phytochemistry.

[82] Armstrong J., Armstrong W. (1999). *Phragmites* die-back: Toxic effects of propionic, butyric and caproic acids in relation to pH. New Phytol..

