# Supplementary material for: The Dynamics of Allelochemicals and Phytotoxicity in Eisenia fetida during the Decomposition of Eucalyptus grandis Litter
Source: Plants (Basel). 2024 Aug 29;13(17):2415. doi: 10.3390/plants13172415 (PMC11397289; doi:10.3390/plants13172415)
Supplement: Supplementary file 1 [file plants-13-02415-s001.zip › plants-3153919-supplementary.pdf]

Supplementary Information

The Dynamics of Allelochemicals and Phytotoxicity in *Eisenia fetida* during the Decomposition of *Eucalyptus grandis* Litter

Contents of this file

Table S1 to S4

Figure S1 to S4

Table S1. Effects of litter extracts from two aged *E. grandis* stands during decomposition on the survival rate of *E. fetida* after 7 and 14 days of exposure.

Decomposition time (Days)	Dose	Survival rate of *E.fetida*(%)			
		4a		8a	
		Exposure 7d	Exposure 14d	Exposure 7d	Exposure 14d
0	CK	100.00±0.00	100.00±0.00	100.00±0.00	100.00±0.00
	C1	93.33±11.55	83.33±28.87	100.00±0.00	93.33±11.55a
	C2	73.33±11.55	50.00±10.00b	93.33±11.55	80.00±26.46
	C3	93.33±5.77	76.67±15.28	96.67±5.77	80.30±10.46
	C4	93.33±5.77	93.33±5.77	90.00±10.00	60.00±34.64
10	CK	96.67±5.77	90.00±10.00	96.67±5.77	90.00±10.00
	C1	100.00±0.00	93.33±5.77	96.67±5.77	86.67±15.28a
	C2	93.33±5.77	86.67±15.28a	100.00±0.00	96.67±5.77
	C3	100.00±0.00	93.33±11.55	96.67±5.77	90.00±10.00
	C4	96.67±5.77	96.67±5.77	96.67±5.77	96.67±5.77
20	CK	73.33±15.28	40.00±17.32	73.33±15.28	40.00±17.32
	C1	86.67±5.77	83.33±5.77	66.67±20.82	40.00±20.00b
	C2	83.33±5.77	76.67±11.55ab	86.67±5.77	83.33±5.77
	C3	76.67±20.82	53.33±23.09	96.67±5.77	96.67±5.77
	C4	90.00±10.00	76.67±15.28	90.00±10.00	76.67±23.09
30	CK	93.33±5.77	83.33±15.28	93.33±5.77	83.33±15.28
	C1	100.00±0.00	96.67±5.77	96.67±5.77	93.33±11.55a
	C2	93.33±11.55	83.33±5.77a	90.00±17.32	80.00±26.46
	C3	96.67±5.77	86.67±15.28	96.67±5.77	80.00±10.00
	C4	96.67±5.77	73.33±25.17	80.00±10.00	66.67±15.28
45	CK	76.67±5.77	63.33±11.55	76.67±5.77	63.33±11.55
	C1	90.00±0.00	60.00±20.00	90.00±17.32	63.33±15.28ab
	C2	96.67±5.77	90.00±10.00a	80.00±10.00	70.00±0.00
	C3	73.33±11.55	60.00±20.00	80.00±20.00	56.67±25.17
	C4	86.67±11.55	83.33±5.77	93.33±5.77	70.00±26.46

Lowercase letters indicate significant differences in decomposition time, $P < 0.05$; mean ± standard error (SE).

Table S2. Effects of litter extracts from two aged *E. grandis* stands during decomposition on the weight inhibition rate of *E. fetida* after 7 and 14 days of exposure.

Decomposition time (Days)	Dose	Weight inhibition rate of *E.fedita*(%)			
		4a		8a	
		Exposure 7d	Exposure 14d	Exposure 7d	Exposure 14d
0	CK	21.60±5.86	33.41±8.66	21.60±5.86	33.41±8.66
	C1	27.66±6.45	29.15±0.05	21.47±1.63	32.38±2.57*
	C2	29.51±8.92	36.56±16.54	23.41±12.02	23.34±3.70
	C3	20.91±4.00b	32.46±4.99b*	16.97±2.40	28.54±2.42*
	C4	18.22±2.66	25.14±6.35	24.54±15.86	30.82±13.95
10	CK	21.85±2.24	44.41±1.65(a)*	21.85±2.24	44.41±1.65*
	C1	25.07±5.22	43.68±2.10(a)*	23.08±9.34	36.00±17.34
	C2	30.22±8.44	45.17±4.72(a)	23.25±2.73	37.49±2.55*
	C3	20.30±5.30b	28.69±5.20b(b)	21.55±12.49	28.63±20.56
	C4	20.46±3.58	28.06±3.93(b)	24.00±3.86	40.41±11.85
20	CK	32.92±4.51(a)	50.18±2.20(a)*	32.92±4.51	50.18±2.20*
	C1	21.06±3.64(bc)	36.88±3.53(b)*	31.53±11.57	35.13±0.19*
	C2	20.12±4.90(c)	37.12±5.70(b)*	17.88±2.18	29.36±4.26
	C3	30.89±1.88a(ab)	54.37±3.04a(a)*	17.74±2.17	25.35±5.58
	C4	20.73±3.20(c)	27.28±3.81(b)	22.59±7.26	34.79±12.53
30	CK	21.08±6.67	26.43±2.62	21.08±6.67	26.43±2.62
	C1	20.02±2.83	33.47±3.72*	22.55±4.02	38.48±2.97*
	C2	15.87±6.44	25.55±0.26*	27.40±4.43	43.19±12.55
	C3	18.93±1.98b	28.66±7.89b	19.55±1.59	35.11±5.16*
	C4	19.37±3.61	33.43±13.31	21.74±3.84	41.96±4.06*
45	CK	31.52±4.38	44.30±13.86	31.52±4.38(a)	44.30±13.86
	C1	28.02±2.93	46.82±3.52*	24.06±1.40(b)	41.12±5.46*
	C2	27.66±4.97	33.29±3.51	21.94±0.48(b)	32.18±8.39*
	C3	24.84±2.60ab	32.99±7.09b	23.99±3.59(b)	38.26±6.97*
	C4	22.71±5.29	32.42±5.96	24.98±1.98(ab)	32.68±8.38

Lowercase letters indicate significant differences with decomposition time, lowercase letters in brackets indicate significant differences among various concentrations, * indicates significant differences between different exposure times, $P < 0.05$; Mean ± standard error (SE).

Table S3. Correlations between the first two axes and environmental factors in RDA and the determination coefficient (r^2) and significance test (Pr) of the correlation between environmental factors and differential potential allelochemicals using the envfit function (R package vegan).

Initial					After 2 months				
Environmental factors	RDA1	RDA2	r^2	Pr(>r)	Environmental factors	RDA1	RDA2	r^2	Pr(>r)
Soil pH	-0.9686**	-0.2485	0.9088	0.0264*	Soil pH	0.8898*	-0.4564	0.7454	0.1458
Soil water content	-1.0000***	-0.0038	0.9827	0.0194*	Soil water content	0.9287**	-0.3709	0.8588	0.0611
Soil organic matter	0.9905***	-0.1377	0.9595	0.0528	Soil organic matter	-0.9549**	0.2970	0.8375	0.0944
Soil total N	0.9968***	-0.0795	0.9473	0.0833	Soil total N	-0.9475**	0.3198	0.8741	0.0458*
Soil available P	0.9964***	-0.0854	0.9569	0.0750	Soil available P	-0.9469**	0.3215	0.8545	0.0639
Shrub	0.9958***	-0.0913	0.9271	0.0833	Shrub	-0.9837***	0.1796	0.9001	0.0278*
Herbaceous	0.2240	-0.9746***	0.2070	0.7792	Herbaceous	-0.6623	-0.7493	0.7895	0.1667
Soil macrofauna	0.9991***	-0.0427	0.5660	0.3347	Soil macrofauna	-0.8460*	0.5331	0.3798	0.4889
Soil microfauna	0.9958***	-0.0921	0.7905	0.0806	Soil microfauna	-0.8119*	0.5838	0.7028	0.1986
Soil microbial	-0.9133*	0.4073	0.5001	0.3528	Soil microbial	1.0000***	0.0064	0.6862	0.1569

*, $P < 0.05$, **, $P < 0.01$, ***, $P < 0.001$; $r_{0.05(4)} = 0.811$， $r_{0.01(4)} = 0.917$， $r_{0.001(4)} = 0.974$.

Table S4. Correlations between the first two axes and differential potential allelochemicals in RDA, and the determination coefficient (r^2) and significance test (Pr) of the correlation between differential potential allelochemicals and growth and physiological properties of *E. fetida* using the envfit function (R package vegan).

Age4					Age8				
Allelochemical	RDA1	RDA2	r^2	Pr(>r)	Allelochemical	RDA1	RDA2	r^2	Pr(>r)
Phenolic acids	-0.9779***	-0.2091	0.9676	0.0292*	Phenolic acids	-0.6210	-0.7838	0.6166	0.3472
Flavonoids	-0.9313**	-0.3643	0.9744	0.0681	Flavonoids	-0.6760	-0.7369	0.6801	0.2042
Terpenoids	-0.9264**	-0.3766	0.9648	0.0889	Terpenoids	-0.6939	-0.7201	0.6552	0.2944
Alkaloids and non-protein amino acids	0.9266**	0.3760	0.9906	0.0083**	Alkaloids and non-protein amino acids	0.7187	0.6954	0.5532	0.4097
Amines	-0.9862***	-0.1655	0.9204	0.0347*	Amines	-0.3220	0.9467**	0.7863	0.0125*

*, $P < 0.05$, **, $P < 0.01$, ***, $P < 0.001$; $r_{0.05(4)} = 0.811$， $r_{0.01(4)} = 0.917$， $r_{0.001(4)} = 0.974$。

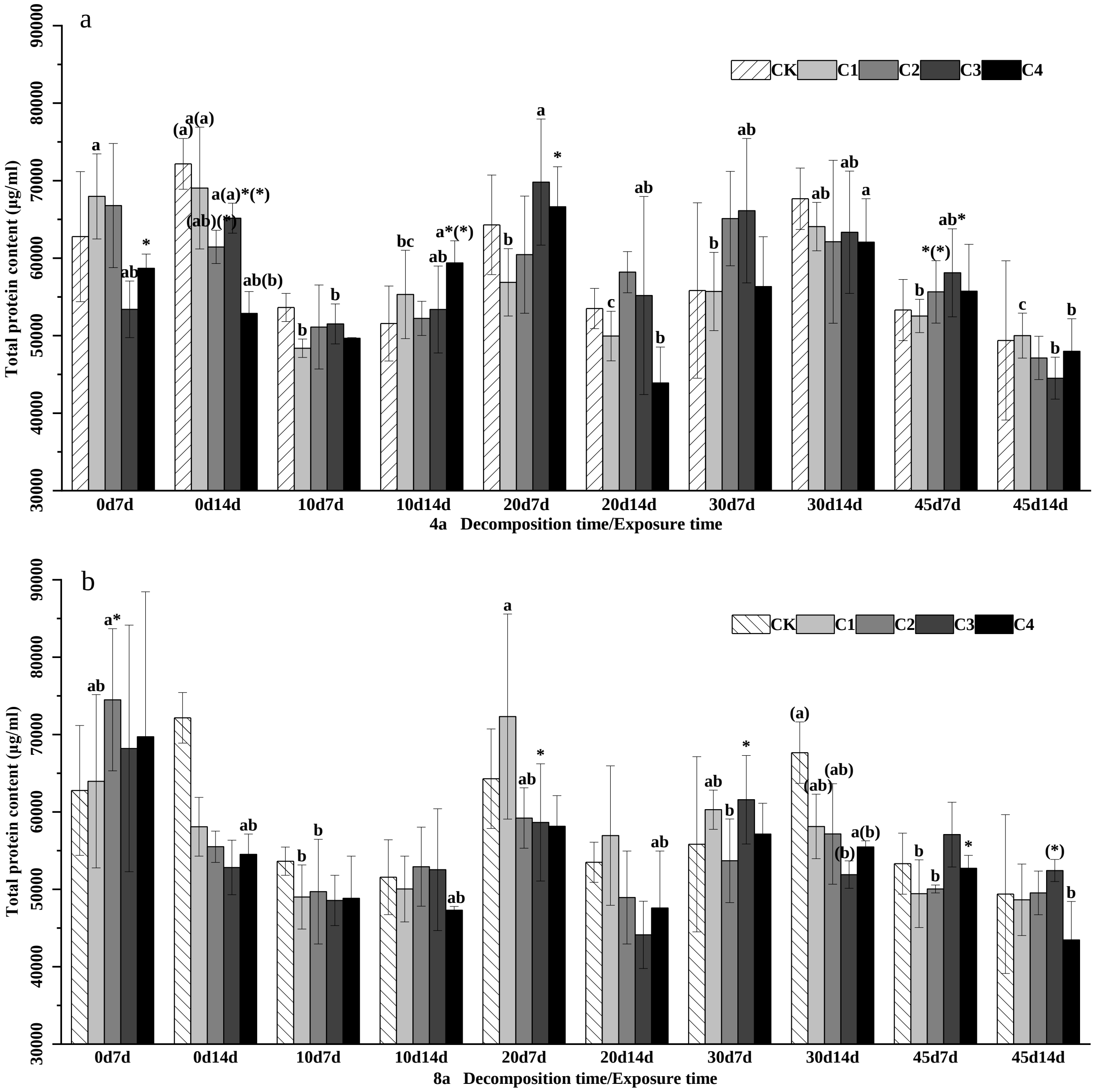

Figure S1. Effects of litter extracts from two mature *E. grandis* stands during decomposition on the total protein content of *E. fetida* after 7 and 14 days of exposure. Lowercase letters indicate significant differences with decomposition time, lowercase letters in parenthesis indicate significant differences with concentrations, * indicates significant differences between the two exposure times, (*) indicate significant differences between the two mature *E. grandis* stands, $P < 0.05$, and error bars indicate standard errors.

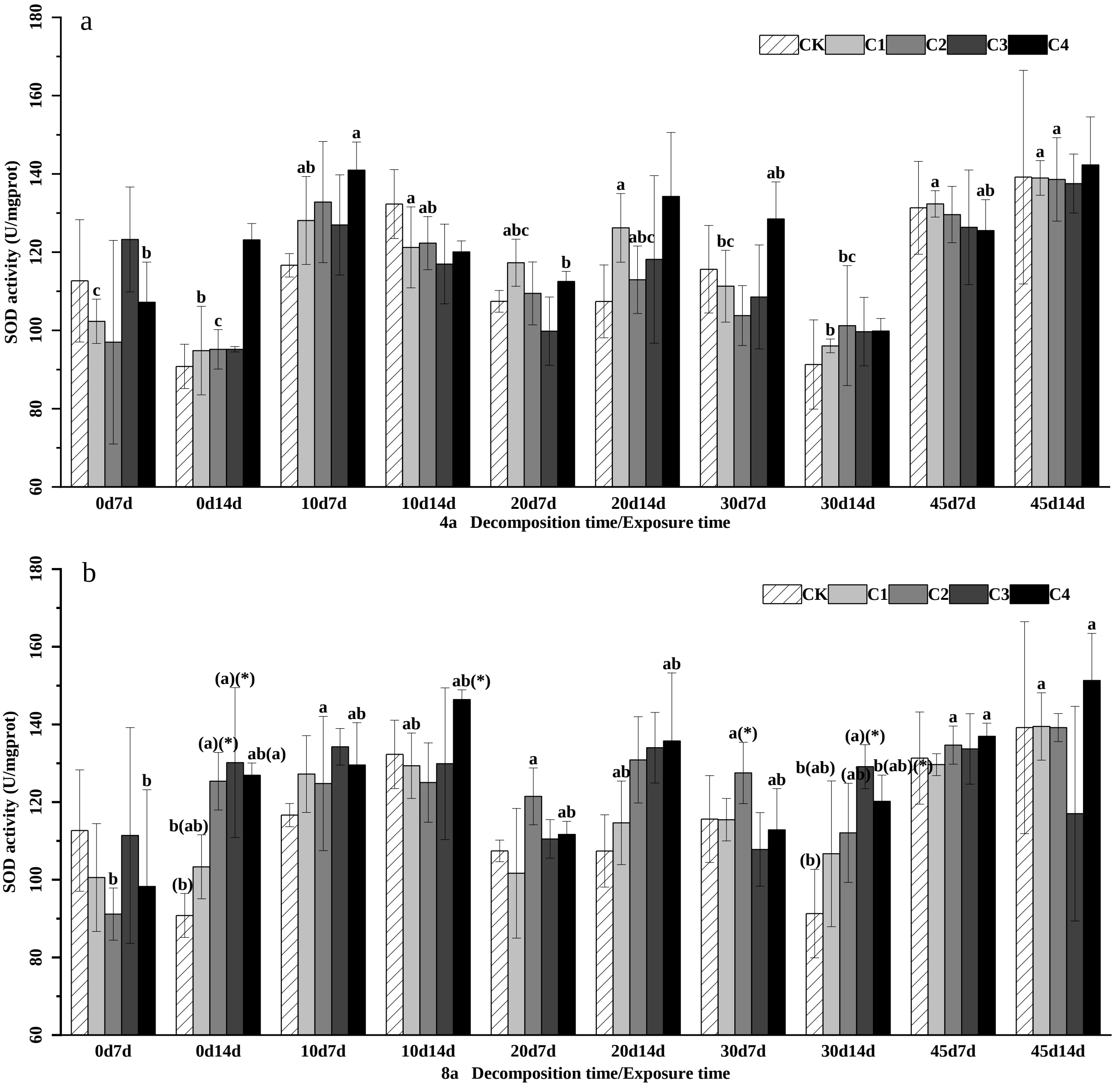

Figure S2. Effects of litter extracts from two mature *E. grandis* stands during decomposition on SOD activity of *E. fetida* after 7 and 14 days of exposure. Lowercase letters indicate significant differences with decomposition time, lowercase letters in parentheses indicate significant differences with concentrations, (*) indicate significant differences between the two aged *E. grandis* stands, $P < 0.05$, and error bars indicate the standard errors.

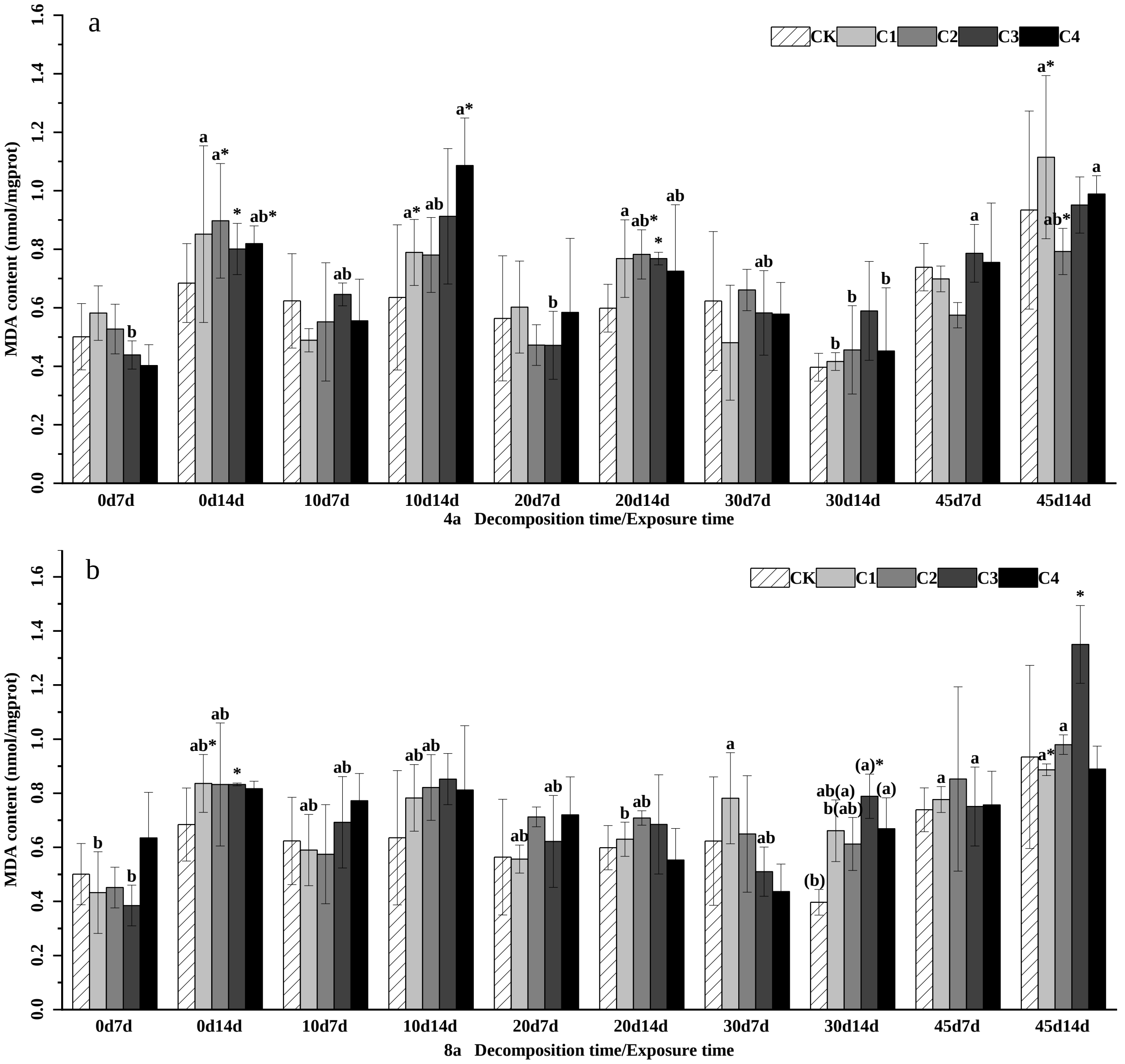

Figure S3. Effects of litter extracts from two mature *E. grandis* stands during decomposition on MDA content of *E. fetida* after 7 and 14 days of exposure. Lowercase letters indicate significant differences with decomposition time, lowercase letters in parenthesis indicate significant differences with concentrations, * indicate significant differences between the two exposure times, $P < 0.05$, and error bars indicate standard errors.

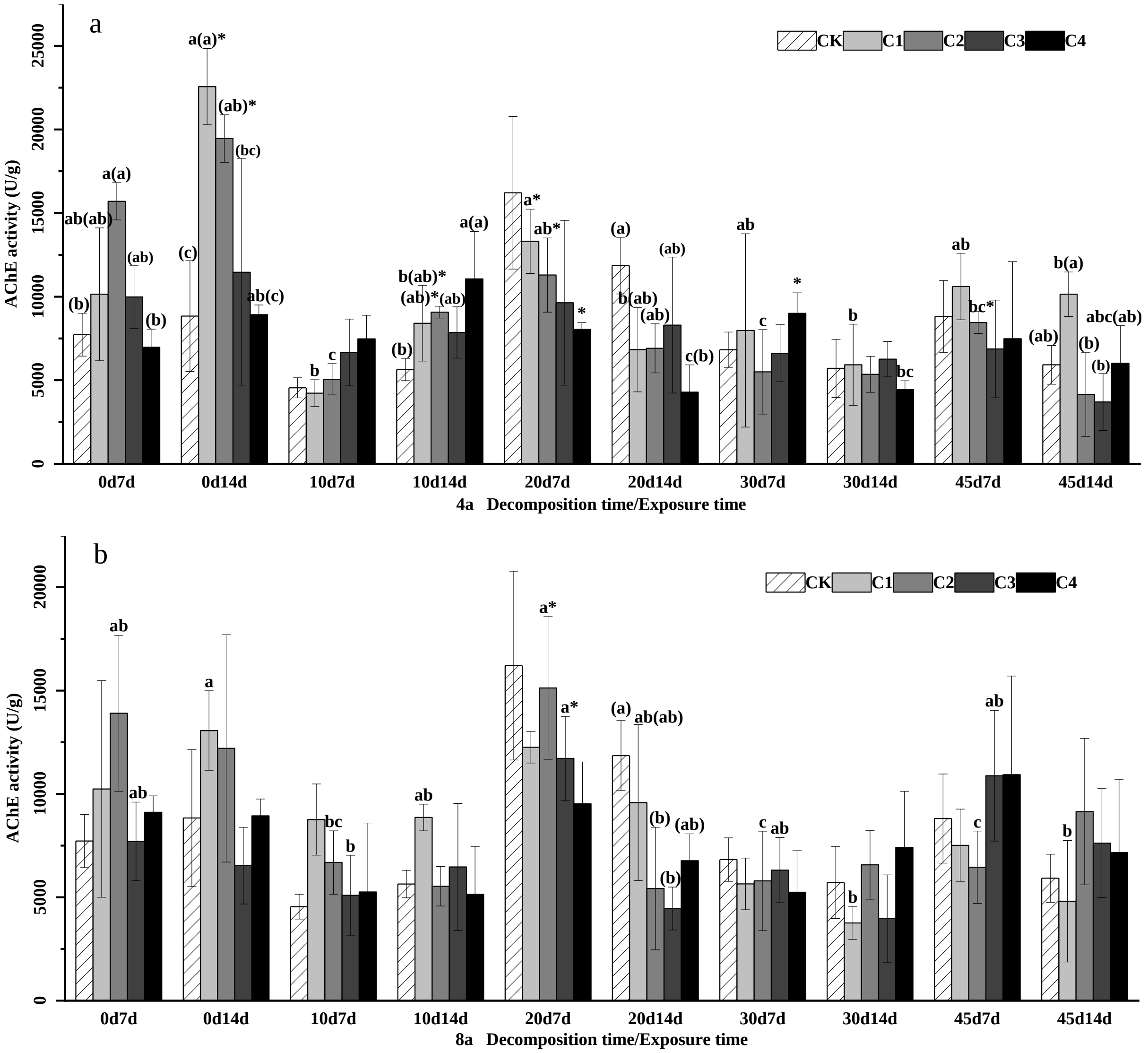

Figure S4. Effects of litter extracts from two mature *E. grandis* stands during decomposition on AChE activity of *E. fetida* after 7 and 14 days of exposure. Lowercase letters indicate significant differences with decomposition time, lowercase letters in parentheses indicate significant differences with concentrations, * indicates significant differences between the two exposure times, $P < 0.05$, and error bars indicate the standard errors.
